# Exploring the Photocyclization Pathways of Styrylthiophenes in the Synthesis of Thiahelicenes: When the Theory and Experiment Meet

**DOI:** 10.1021/acs.joc.1c00147

**Published:** 2021-03-26

**Authors:** Bianca C. Baciu, José Antonio Vergés, Albert Guijarro

**Affiliations:** † Departamento de Química Orgánica and Instituto Universitario de Síntesis Orgánica, Campus de San Vicente del Raspeig, Universidad de Alicante, Apdo. 99, 03080 Alicante, Spain; ‡ Departamento de Teoría y Simulación de Materiales, Instituto de Ciencia de Materiales de Madrid (CSIC), Cantoblanco, 28049 Madrid, Spain

## Abstract

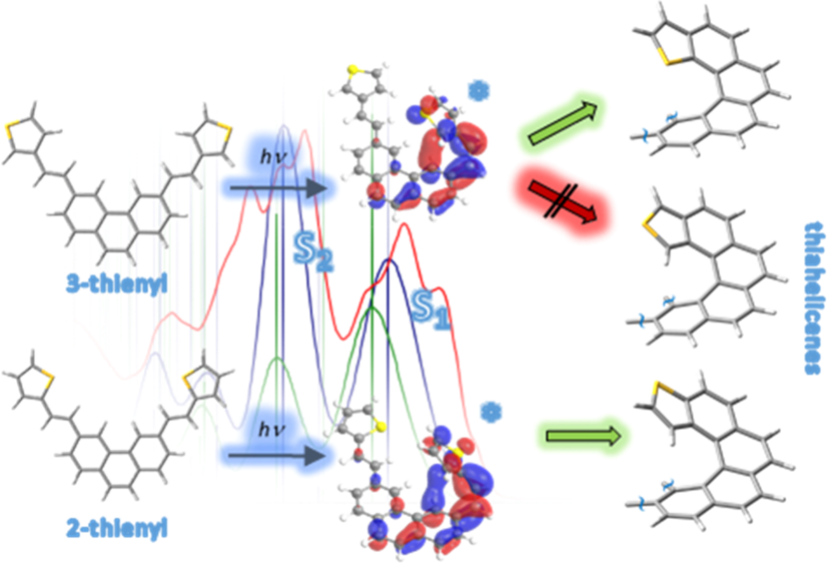

The introduction of thiophene rings to the helical structure of carbohelicenes has electronic effects that may be used advantageously in organic electronics. The performance of these devices is highly dependent on the sulfur atom topology, so a precise knowledge of the synthetic routes that may afford isomeric structures is necessary. We have studied the photocyclization pathway of both 2- and 3-styrylthiophenes on their way to thiahelicenes by experiment and theory. To begin with, the synthesis of stereochemically well-defined 2- and 3-styrylthiophenes allowed us to register first, and simulate later, the UV–vis electronic spectra of these precursors. This information gave us access through time-dependent density functional theory calculations to the very nature of the excited states involved in the photocyclization step and from there to the regio- and stereochemical outcome of the reaction. For the widely known case of a 2-styrylthiophene derivative, the expected naphtho[2,1-*b*]thiophene type of ring fusion was predicted and experimentally observed by synthesis. On the contrary, 3-styrylthiophene derivatives have been seldom used in synthetic photocyclizations. Among the two possible structural outcomes, only the naphtho[1,2-*b*]thiophene type of ring fusion was found to be mechanistically sound, and this was actually the only compound observed by synthesis.

## Introduction

**Scheme 1:**
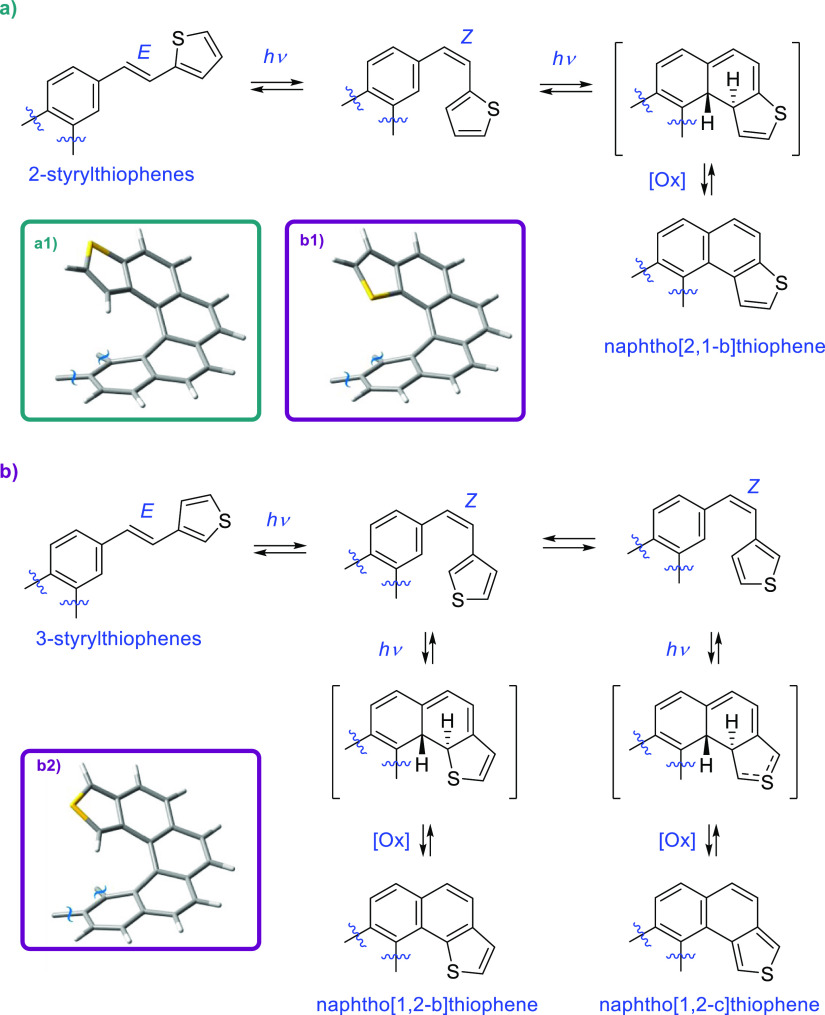
Reaction Pathways for (a) 2- and (b) 3-Styrylthiophenes Based on the Widely Accepted Mechanism of Photocyclization Inset (a1) framed in green shows the 3D view of a thiophene-terminated helicenic structure originated from reactions in (a), while (b1) and (b2) framed in magenta would originate from reactions in (b).

Helicenes display a robust, fully conjugated helical architecture that makes them prototypes of chiral carbon nanostructures, with increasing applications in organic electronics.^1^ With the inclusion of thiophene rings into the helical structure, two noticeable effects occur. First, a stable doped state with improved conducting properties arises;^2,3^ second, an enhanced binding energy with metallic electrodes results.^3−5^ Both are the consequence of an enriched π-electron density induced by the sulfur atom. These two aspects are highly dependent on the sulfur atom position and bond topology generated therefrom within the thiahelicene,^6,7^ so synthetic routes must be explicit in that respect. In their seminal studies, Wynberg and coworkers used the photocyclization of 2-styrylthiophene derivatives as a straightforward way to access to thiahelicenes (Scheme 1a).^8^ Developed half a century ago, it remains a prevalent, still captivating method to achieve the type of ring fusion needed to construct helicenes starting from one of the simplest conceivable precursors, so a large number of thiophene-containing helicene structures have been obtained this way since then.^9^ The general accepted mechanism does not differ much from the classical Mallory reaction of stilbenes^10^ and involves a fast *Z*–*E* isomerization of the styrylthiophene precursor,^11^ followed by a key photocyclization step, namely, photochemical electrocyclic reaction, to afford 9*a*,9*b*-dihydronaphthothiophenes as transient intermediates,^12^ which in turn dehydrogenate in the reaction media by means of an oxidizer to afford the aromatic naphthothiophene moiety (Scheme 1a). It became already apparent at that early stage that 3-styrylthiophenes were far less prolific precursors (Scheme 1b) to the point that there are barely no examples of this photocyclization strategy in the synthesis of thiahelicenes up to until very recently. Although highly under-represented, it is a valuable alternative for the construction of unusual thiophene-terminated helicenes. Focusing on the central features of this photocyclization and particularly on its backbone scaffold (Scheme 1b), it has been reported to afford not only naphtho[1,2-*b*]thiophenes^13^ but also its isomeric naphtho[1,2-*c*] counterpart,^14^ which may leave doubts about the actual natural outcome of this reaction. This is a critical yet obscure point that gives rise to different topologies in the sulfur atom positioning, and with it, to the entire electronic structure of the corresponding thiahelicene. This is an important matter for many reasons, but in particular, if the objective of the synthesis is to build conductive yet robust nanocontacts between a helicene and external metallic electrodes. It is known that quantum interference^15^ may lead to constructive or destructive interference, enhancing or suppressing conductance depending on the sulfur atom positioning. Within this scenario, we decided to study in depth this photocyclization in the framework of our studies pursuing different thiophene-terminated helicenes with special regard to the topology originated from the sulfur atom positioning in the key photochemical step. In line with those aims, this work is a combined experimental-theoretical study structured as follows: (a) first, we synthesized suitable 2- and 3-styrylthiophenes as stereochemically homogeneous precursors of thiahelicenes, and then, (b) we studied both experimentally and theoretically their absorption process (excitation) and subsequent key photocyclization step. Finally, (c) we isolated the corresponding thiahelicenes as reaction products and characterized them unequivocally to establish the proper reaction pathway. We decided to work with thiophene doubly terminated helicenes (or more specifically with dithiahelicenes **1** and **2**; Scheme 3) throughout all this mechanistic study for practical reasons, in line with our developing project on molecular solenoids. Their applications as single-molecule devices in organic electronics are currently under study.

## Results and Discussion

### Synthesis of (*E,E*)-3,6-bis(2-(Thiophen-2-yl)vinyl)phenanthrene (**7**) and (*E,E*)-3,6-bis(2-(Thiophen-3-yl)vinyl)phenanthrene (**8**) as Stereochemically Well-Defined Photochemical Precursors

**Scheme 2:**
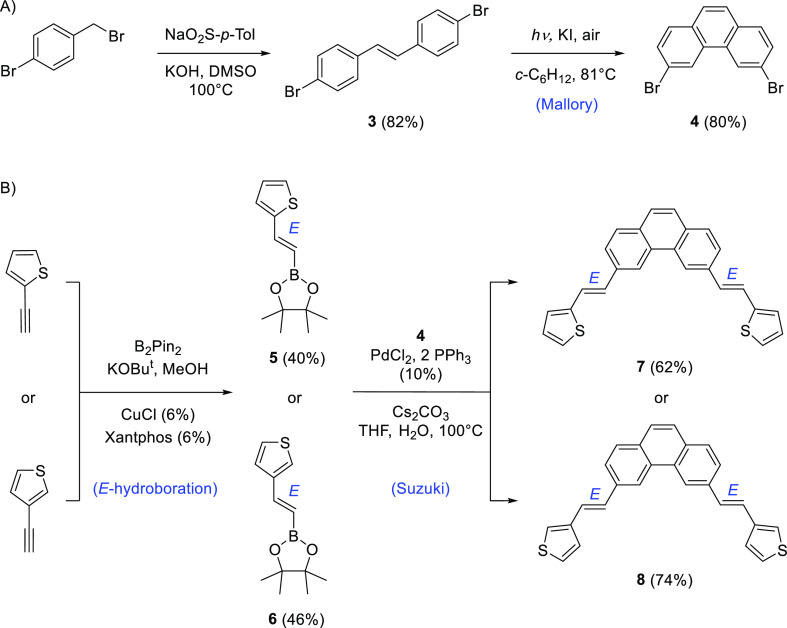
Synthesis of All-*trans* (*i.e.*, Configurationally Homogeneous) (*E*,*E*)-3,6-bis(2-(Thiophen-2-yl)vinyl)phenanthrene (**7**) and (*E*,*E*)-3,6-bis(3-(Thiophen-2-yl)vinyl)phenanthrene (**8**)

Stereochemically well-defined 2- and 3-styrylthiophene precursors were required to trace the following key photochemical step of the synthesis. In the simplest approach, the synthesis of an all-*trans* configuration of the double bonds was sought. It was carried out according to Scheme 2. First, the central symmetric fragment 3,6-dibromophenanthrene (**4**) was prepared from 4,4′-dibromostilbene (**3**) by means of a Mallory reaction.^16^ Stilbenic precursor **3** was easily prepared in one step from commercial 4-bromobenzyl bromide taking advantage of the dual electrophilic-nucleophilic role of this reagent in the presence of sodium *p*-toluenesulfinate under basic conditions (KOH).^17^ In situ elimination afforded *trans*-stilbene **3** in a good yield, which was photocyclized very efficiently using a 400 W high-pressure Hg lamp (see the Supporting Information) to **4**. Next, two arms containing the desired *E*-2- and *E*-3-thienylvinyl fragments, respectively, were attached to central fragment **4***via* a Suzuki coupling, maintaining the stereochemical integrity of *E*-vinylborane reagents **5** and **6** chosen for this purpose. These last reagents were conveniently prepared with a well-defined stereochemistry through a room-temperature CuCl-xantphos-catalyzed hydroboration of the commercially available acetylenic starting materials using bis(pinacolato)diboron (B_2_Pin_2_) and potassium *t*-butoxide in methanol.^18^

### Understanding the Photochemical Reaction Pathway

#### Experimental UV–Vis Spectra of (*E,E*)-3,6-bis(2-(Thiophen-2-yl)vinyl)phenanthrene (**7**) and (*E,E*)-3,6-bis(2-(Thiophen-3-yl)vinyl)phenanthrene (**8**)

**Figure 1:**
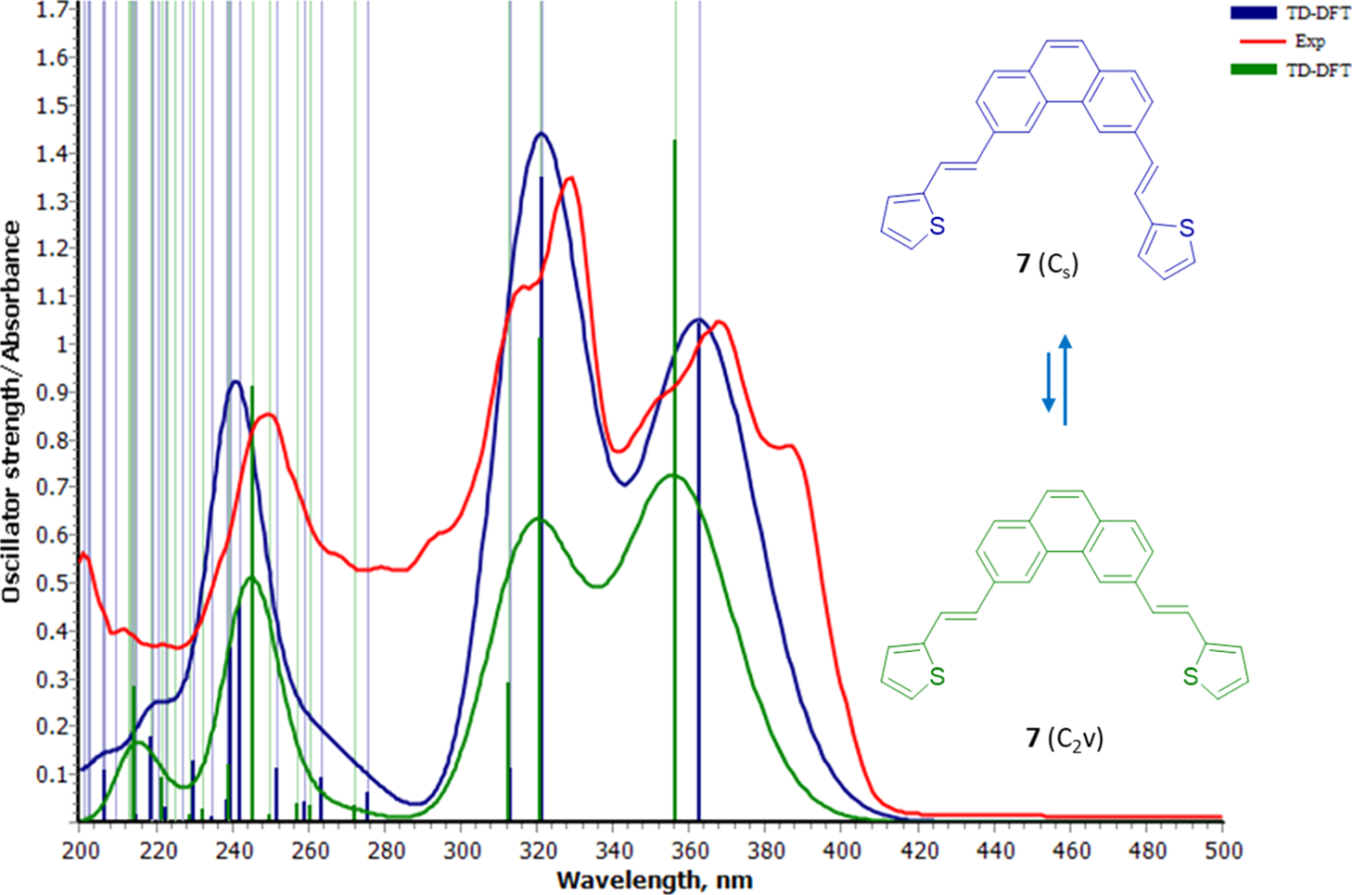
Experimental UV–vis spectrum of compound **7** (red line) in *n*-hexane. Calculated spectra of the most abundant conformer **7**(C_s_) (blue line) and second most abundant **7**(C_2v_) (green line, with its intensity halved, see the text) superimposed, as well as the calculated transitions (vertical lines) and oscillator strengths (thick vertical lines).

**Figure 2:**
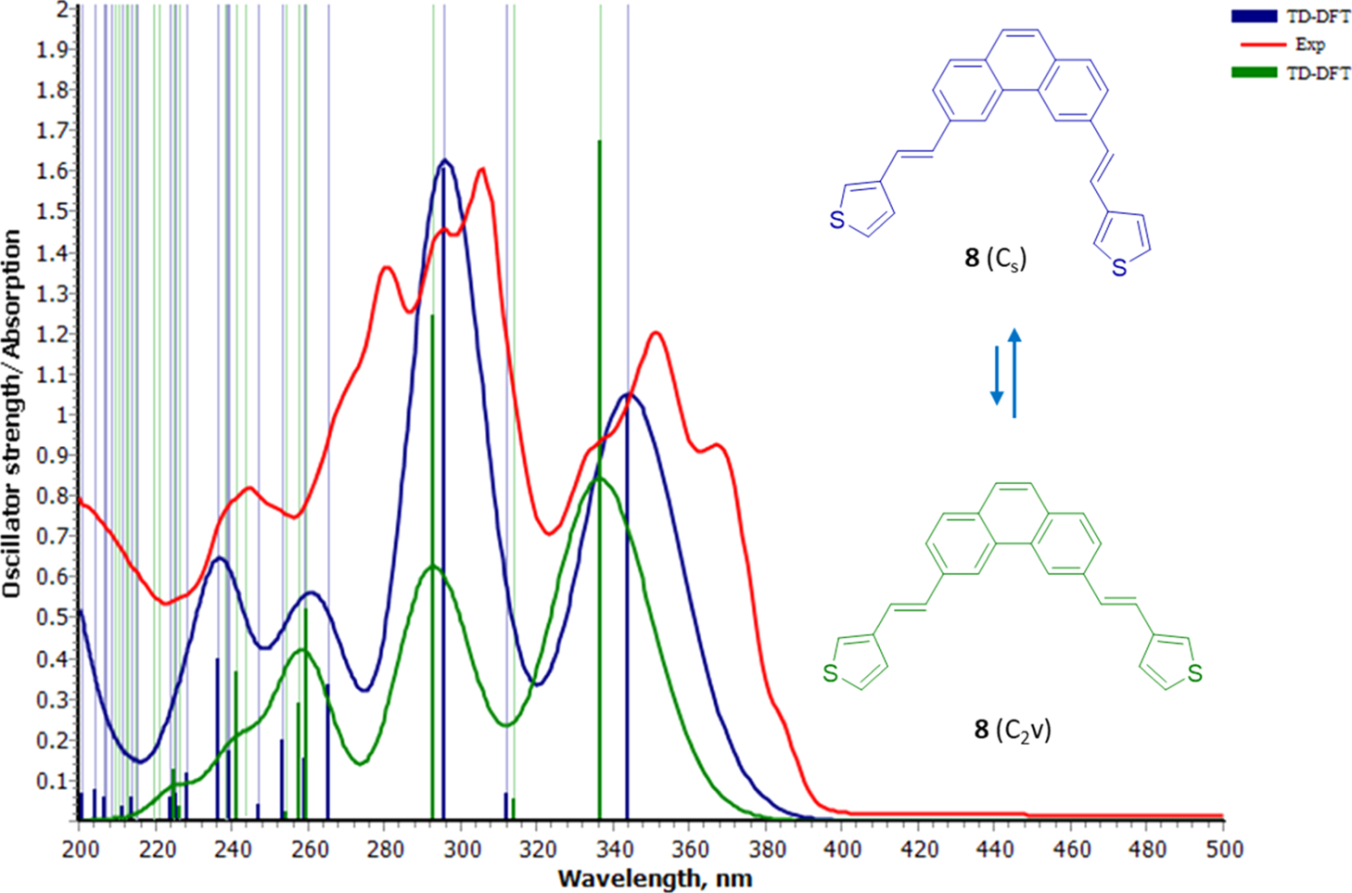
Experimental spectrum of compound **8** (red line) in *n*-hexane. Calculated spectra of the most abundant conformer **8**(C_s_) (blue line) and second most abundant **8**(C_2v_) (green line, with its intensity halved, see the text) superimposed, as well as the calculated transitions (vertical lines) and oscillator strengths (thick vertical lines).

Styrylthiophene compounds **7** and **8** are the photochemical precursors of the targeted dithia[7]helicenes **1** and **2** in this study. The experimental UV–vis spectra of **7** and **8** in *n*-hexane are included in Figures 1 and 2 (red lines, see also the Supporting Information). In both cases, the absorption patterns share many similarities. For 2-thienylstyryl derivative **7**, it consists of three main absorptions, the first centered at λ_max1_ = 368.0 nm, with a side peak at 385.8 nm (*i.e.*, 1253.7 cm^–1^ away from it) and a nearly symmetrical shoulder at higher energy evidencing a vibrational fine structure. There is another absorption at λ_max2_ = 328.1 nm, also with a shoulder at slightly higher energy, and additional absorptions at lower wavelengths, for example, λ_max3_ = 250.0 nm, less relevant for us since they are out of the reach of the reactor lamp.

For 3-thienylstyryl derivative **8**, the spectrum also consists of three main absorptions, the first two displaying a clear vibrational fine structure again. The first band centered at λ_max1_ = 350.9 nm has a side peak at 366.9 nm (*i.e.*, 1242.7 cm^–1^ away from it) and also a nearly symmetrical shoulder at higher energy. A second main absorption occurs at λ_max2_ = 306.0 nm, with two consecutive well-resolved vibrational peaks at 295.5 and 280.7 nm, and an additional smaller band at λ_max3_ = 244.4 nm that is nonrelevant for our purposes, complete the experimental spectrum.

With this information in hands, we calibrated our quantum chemistry methods and began the theoretical simulation of the electronic spectrum.

### Conformational Equilibria of **7** and **8**

First, we studied the conformational equilibria of **7** and **8**. There are four single bonds in each molecule, giving rise to a quite significant amount of 2^4^ = 16 conformations by rotation, which finally get reduced by symmetry arguments to 10 nonequivalent molecular conformations (only flat, fully delocalized structures were taken into account at this stage). A complete account of all the conformers, their energies, and vibrational analysis can be found in the Supporting Information. From them, a Maxwell–Boltzmann distribution reveals two prevailing conformations very close in energy (<0.4 kcal/mol, see the Supporting Information) that stand out, accounting for more than 60% of the overall conformational population. They are represented in Figures 1 and 2 (inset).

The calculated spectral shapes of **7**(C_s_) and **8**(C_s_) fit reasonably well with the experimental spectra, reproducing λ_max1_, λ_max2_, and somewhat less the more complex band at λ_max3_, as well as the intensities of their absorbances. Their populations are doubled with respect to the minor C_2v_ conformers since they have degenerated structures and benefit from the statistical effect of a smaller symmetry number (C_s_ compared to the C_2v_ point group).^19^ For the minor conformers **7**(C_2v_) and **8**(C_2v_), the calculated transitions are of similar energy to the C_s_ ones, while their intensities are mismatched (green vs blue oscillator strengths, vertical thick lines, and overall calculated spectral shapes). Thus, by comparison with the experimental spectrum, it is evident that the statistical effect due to the symmetry overcomes the rather negligible difference in energy calculated between both conformers. We halved the simulated spectrum for C_2v_ conformers in Figures 1 and 2 to stress this argument. We have therefore identified the transitions that give rise to the relevant absorption bands at λ_max1_ and λ_max2_, always keeping in mind that the experimental absorptions have a more complex pattern originated from vibronic coupling; this simulation is out of the reach of this work.

### Calculated Electronic Spectra of the Major Conformations of **7** and **8**

The assignment of the electronic spectra for the most representative conformations **7**(C_s_) and **8**(C_s_) and minor counterparts **7**(C_2v_) and **8**(C_2v_) calculated by time-dependent density functional theory (TDDFT) at the wB97XD/6-311++G(2d,2p) level of theory in *n*-hexane as the polarizable continuum model (PCM) is collected in Tables 1 and 2. We confine our discussion to the transitions with oscillator strengths *f* > 0.10 and contributions with more than 10% weight.

**Table 1:** Calculated UV–Vis Spectrum of Major Conformations **7**(C_s_) and **7**(C_2v_). State Number, Wavelength (λ), Energy (*E*), Oscillator Strength (*f*), and Assignment of the Transitions

state	λ (nm)	*E* (eV)	*f*	assignment		
**7**(C_s_)						
S_1_	362.6	3.419	1.04	H → L (78%)	H – 1 → L + 1 (16%)	
S_2_	321.6	3.855	1.35	H – 1 → L (46%)	H → L + 1 (40%)	
S_3_	313.0	3.961	0.11	H → L + 2 (46%)	H – 2 → L (26%)	
S_7_	252.0	4.920	0.11	H – 2 → L (46%)	H → L + 2 (17%)	H – 1 → L (12%)
S_8_	242.0	5.123	0.46	H – 2 → L + 2 (44%)		
S_9_	239.7	5.173	0.36	H – 4 → L (27%)	H – 5 → L + 1 (18%)	H – 4 → L + 1 (16%)
S_12_	229.9	5.392	0.13	H – 1 → L + 2 (21%)	H – 2 → L +1 (21%)	H → L + 10 (13%)
S_18_	219.0	5.662	0.18	H – 1 → L + 1 (28%)	H – 3 → L (25%)	H – 6 → L + 1 (16%)
S_22_	206.71	5.998	0.11	H – 3 → L + 2 (27%)		
**7**(C_2v_)						
S_1_	356.4	3.478	1.43	H → L (77%)	H – 1 →L + 1 (18%)	
S_2_	321.2	3.861	1.01	H → L + 1 (57%)	H – 1 →L (21%)	
S_3_	312.7	3.965	0.29	H → L + 2 (36%)	H – 1 →L (29%)	H – 2 →L (18%)
S_8_	245.4	5.053	0.91	H – 2 → L + 2 (29%)	H – 2 → L + 1 (29%)	H – 1 → L + 1 (21%)
S_9_	239.2	5.183	0.12	H – 5 → L (38%)	H – 4 →L + 1 (33%)	H – 5 → L + 4 (10%)
S_18_	214.3	5.786	0.28	H – 3 → L (22%)	H – 1 →L + 1 (21%)	H – 6 → L + 1 (14%)

**Table 2:** Calculated UV–Vis Spectrum of Major Conformations **8**(C_s_) and **8**(C_2v_). State Number, Wavelength (λ), Energy (*E*), Oscillator Strength (*f*), and Assignment of the Transitions

state	λ (nm)	*E* (eV)	*f*	assignment		
**8**(C_s_)						
S_1_	344.1	3.603	1.05	H → L (82%)	H – 1 → L + 1 (10%)	
S_3_	295.8	4.192	1.61	H → L + 1 (50%)	H – 1 → L (38%)	
S_4_	265.3	4.673	0.34	H – 1 → L + 2 (30%)	H – 2 → L + 2 (16%)	H – 3 →L (10%)
S_5_	259.0	4.787	0.15	H → L + 2 (32%)	H – 2 → L (16%)	H – 1 →L (15%)
S_6_	253.3	4.896	0.20	H – 2 → L + 1 (28%)	H – 2 → L + 2 (13%)	
S_8_	239.1	5.185	0.17	H → L + 8 (21%)	H – 1 → L + 6 (19%)	
S_10_	236.5	5.242	0.40	H – 2 → L + 2 (36%)	H – 1 → L + 1 (27%)	H – 3 →L (12%)
S_11_	228.4	5.429	0.12	H – 2 → L + 1 (28%)	H – 1 → L + 2 (18%)	H – 2 →L + 2 (15%)
S_30_	196.6	6.307	0.38	H – 5 → L + 8 (25%)	H – 4 → L + 6 (19%)	H – 4 →L (11%)
**8**(C_2v_)						
S_1_	336.6	3.684	1.67	H → L (82%)	H – 1 → L + 1 (11%)	
S_3_	292.7	4.236	1.25	H – 1 → L (52%)	H →L + 1 (27%)	H → L + 2 (11%)
S_4_	259.4	4.780	0.52	H – 1 → L + 2 (47%)	H – 2 →L + 2 (18%)	H – 3 → L (11%)
S_5_	257.6	4.813	0.29	H – 2 → L + 1 (39%)	H →L + 12 (16%)	H – 1 → L + 1 (14%)
S_8_	241.2	5.142	0.37	H – 1 → L + 1 (38%)	H – 2 → L + 1 (28%)	H – 3 → L (13%)
S_12_	225.0	5.511	0.13	H – 4 → L (27%)	H – 4 → L + 6 (13%)	H – 5 → L + 1 (11%)

**Figure 3:**
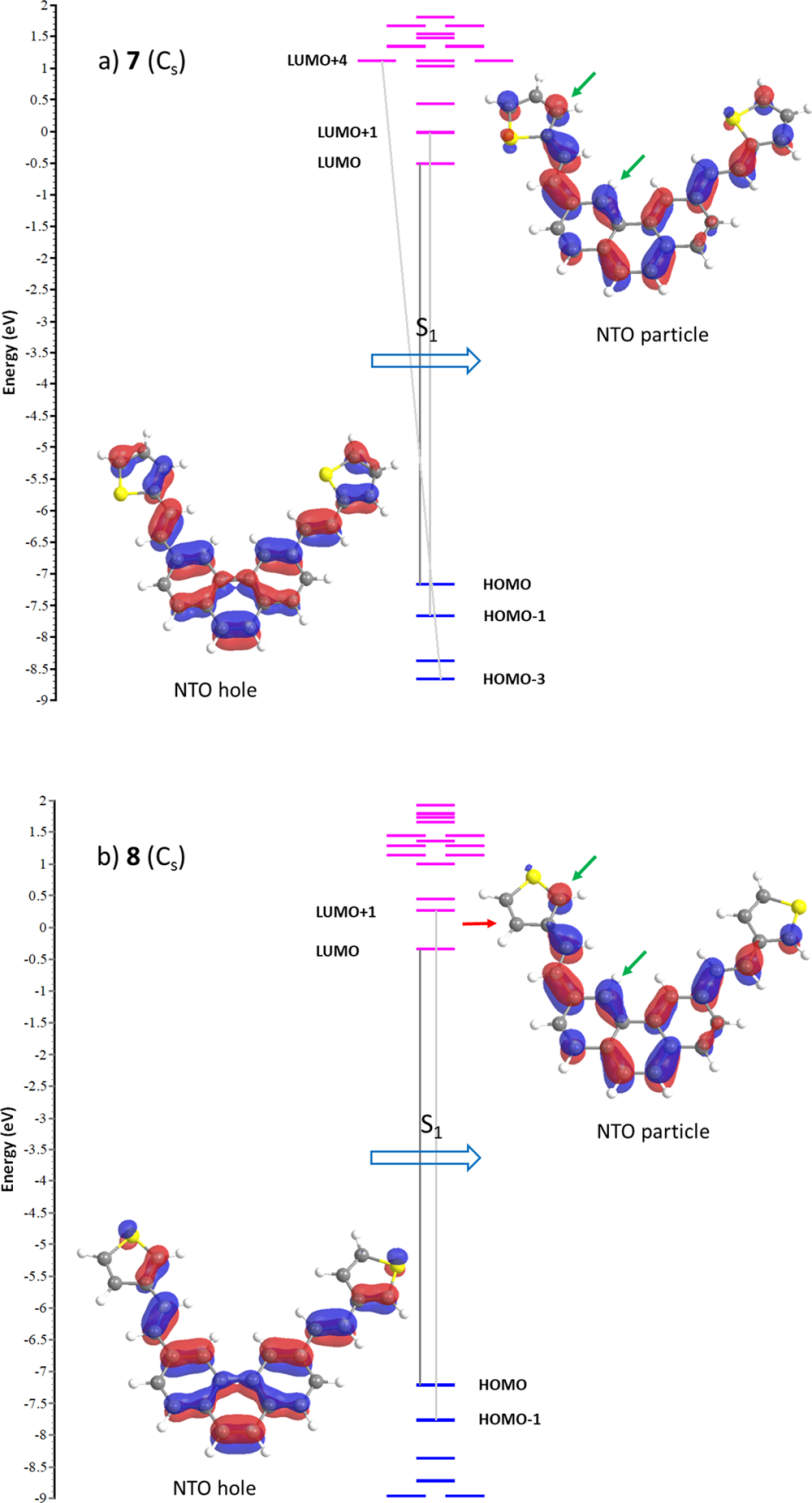
Vertical transition to the first excited state S_1_ for the major conformations of (a) **7**(Cs) and (b) **8**(Cs), represented as the hole and particle NTO pair [in this case, HOMO → LUMO weighing 78 and 82% of the transition for (a,b), respectively], and the diagram of MOs involved in the excited states. Green arrows signal the reacting pair of carbons.

Let us focus now on the key transitions of the simulated spectra. For **7**, the vertical transition to the S_1_ state from the ground state S_0_ for any of its major conformations is a π → −π* type of excitation, mainly described by the highest occupied molecular orbital (HOMO) → lowest unoccupied molecular orbital (LUMO) transition combined with a HOMO – 1 → LUMO + 1 in a much smaller weight along with other even minor unreported contributions (Table 1). In Figure 3a, we can see this transition for the major conformer **7**(C_v_) at λ_1_ = 362.6 nm as a natural transition orbital (NTO),^20^ representing the best hole/particle pair representation of the transition density matrix for that excited state. The MO diagram in the middle shows the electronic configurations that span the actual excited-state wavefunction. The composition of this peak is very similar for both conformers C_s_ and C_2v_ (and indeed for the rest of the less representative conformers studied) and corresponds to the experimental λ_max1_ = 368.0 nm. As can be seen in Figure 3, the binding pair of carbons are the C(4) phenanthrene position and the C(3) of thiophene (green arrows), with a typical *supra-antara* stereochemistry as well as a weakened stilbenic double bond (antibonding interaction). This will enable its dihedral rotation and meeting of the reacting pair of carbons as the reactions further progress in the S_1_ surface. The main characteristics of these NTOs are conserved for both the *E*- and *Z*-isomers, the excited state of which will eventually drive to the expected naphtho[2,1-*b*]thiophene type of ring fusion as we will see later.

The following calculated vertical transition is S_2_, located at λ_2_ = 321.6 nm for the major conformer **7**(C_v_) and nearly the same for the minor, and corresponds to the experimental λ_max2_ = 328.1 nm. A description of the MOs involved in this vertical transition to the S_2_ state for **7** can be found in the Supporting Information. This state also provides an analogous reaction pathway with a regio- and stereochemical outcome akin to the above explained for S_1_. Both S_1_ and S_2_ states can explain the photochemical reaction outcome, although the lowest excited state is usually the relevant one in terms of photochemical production based on half-life arguments (Kasha’s rule,^21^ extended to chemical deactivation).

We now move to the more interesting case **8**. For **8**, again the vertical transition S_0_ → S_1_ for any of its major conformations is a π → −π* type of excitation, mainly described by a HOMO → LUMO transition combined with a HOMO – 1 → LUMO + 1 in a much smaller weight (Table 2). In Figure 3b, we can see this transition for the major conformer **8**(C_v_) at λ_1_ = 344.1 nm as an NTO, along with the MO diagram of the transition. Just as in the former case, the composition of this peak is very similar for both conformers C_s_ and C_2v_ and the rest of the less representative conformers and corresponds to the experimental λ_max1_ = 350.9 nm. It can be deduced from Figure 3b that the binding pair of carbons are the C(4) phenanthrene position and the C(2) of thiophene (green arrows), while the potentially competing C(4) is kinetically unreactive (red arrow) through this excitation pathway. Once evolved to the *Z*-conformation, its excited state will eventually lead to the expected naphtho[1,2-*b*]thiophene type of ring fusion but not to the naphtho[1,2-*c*] one (see also structures in Scheme 1), as we will see later.

The following calculated vertical transition of relevance is S_3_, located at λ_2_ = 295.8 nm for the major conformer **8**(C_v_) and very close for the minor one (C_2v_), and corresponds to the experimental λ_max2_ = 306.0 nm. Similar arguments as those explained before hold in here. A description of the MOs involved in this vertical transition to the S_3_ state for **8** can be found in the Supporting Information. Again, this state also provides an analogous reaction pathway with a regio- and stereochemical outcome akin to the above explained for S_1_.

### Calculated Excited States of **7** and **8** and the Photochemical Reaction Pathway

**Figure 4:**
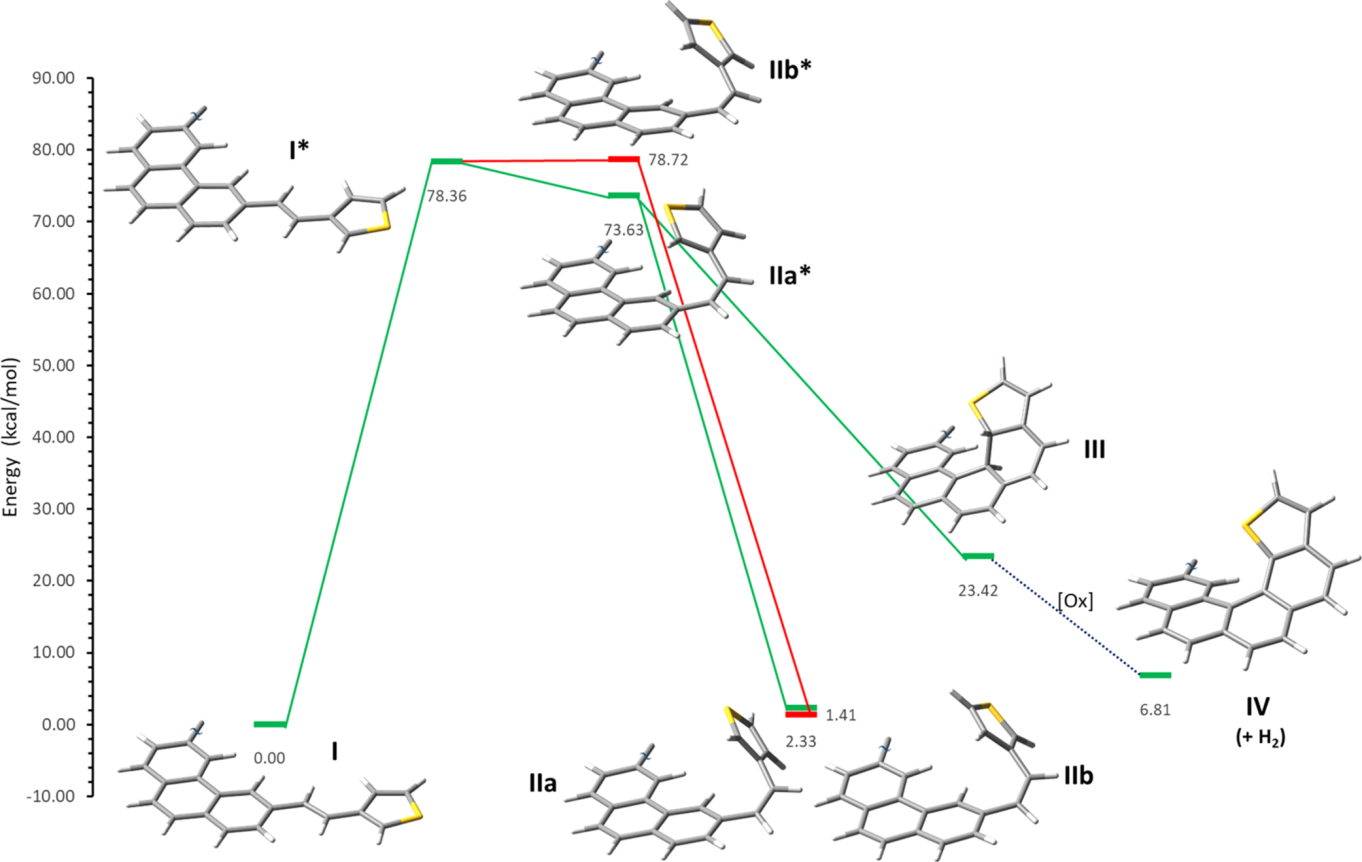
Diagram of relative energy showing the photochemical pathways that **8** (C_2v_) (represented here as **I**) may follow upon S_0_ → S_1_ excitation. Calculated absolute and relative minima located in the ground and excited states (*) are also represented. An allowed route that goes through IIa* leading both to *E* ⇆ *Z* isomerization (**I** ⇆ **IIa**) and to the cyclized product (**I** ⇆ **III**) is shown in green. Conformer IIb* leading only to *E* ⇆ *Z* isomerization (**I** ⇆ **IIb**) is shown in red. No stationary cyclized intermediate was found that could lead to the helicenic final product.

**Figure 5:**
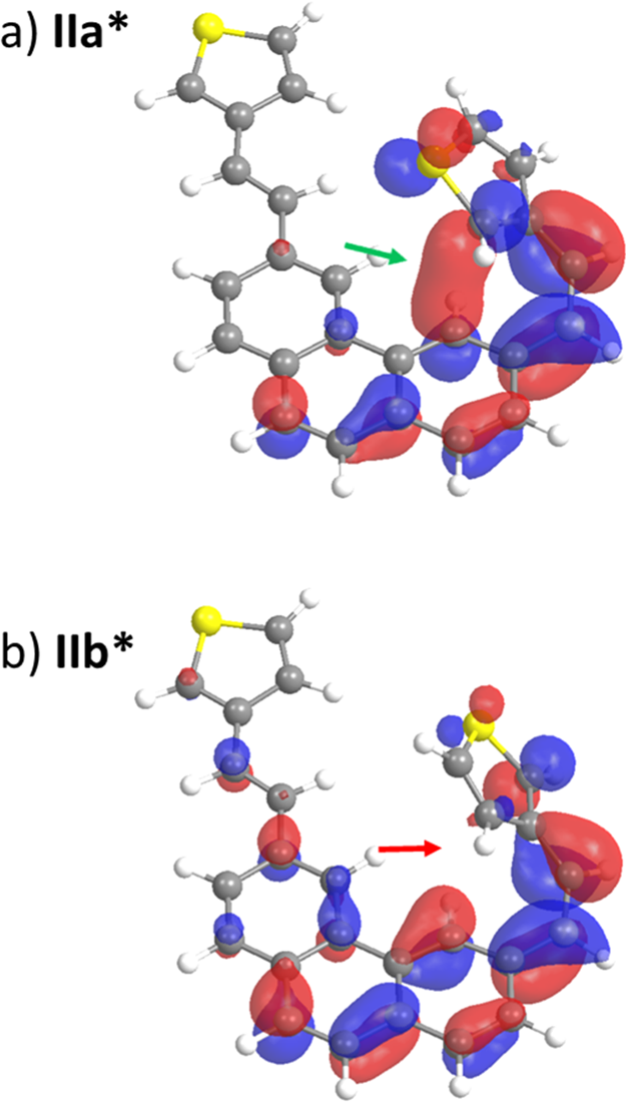
Relevant minima IIa* and IIb* of the first excited state S_1_ of **8** and changes in the electron density represented as the particle NTO. A developing sigma-binding interaction in IIa* (green arrow) and the lack of the same in IIb* (red arrow) is shown.

The above-described vertical transitions may evolve within the excited-state hypersurface to minimal energy conformations and eventually to a suitable excited *Z*-configuration for the cyclization step. We have tracked this evolution in the excited state S_1_ for **7** and **8**, starting from the most symmetric conformations (C_2v_) for simplicity. Calculations were done by density functional theory (DFT) and TDDFT at the wB97XD/6-311++G(2d,2p) level of theory in *n*-hexane as the PCM. Results are collected in Figure 4 and the Supporting Information. Upon vertical excitation, **8** (C_2v_) (**I** in Figure 4) in the first place evolves to a planar excited state I* (relative minimum) that further relaxes to IIa* (absolute minimum in S_1_), while IIb* (also a relative minimum) is some 5.1 kcal/mol above the former. Both are the two relevant conformers of the excited state S_1_ with a twisted but near to *Z*-configuration of the stilbenic double bond. The study of the changes in the electron density upon excitation in IIa* and IIb* by NTO analysis is revealing. IIa* has an important binding interaction between the carbons C(4) of phenanthrene and C(2) of thiophene, which imparts extra stability to this excited state and entails an incipient sigma bond formation (Figure 5a, green arrow). After internal conversion and vibrational relaxation to the ground state, it may lead to the dihydro-intermediate **III** or alternatively the *Z*-isomer **IIa** (Figure 4, green reaction pathway). A typical *supra-antara* stereochemistry and a weakened stilbenic double bond (C–C=C–C dihedral angles of 30 and 31° for IIa* and IIb*, compared to nearly 0° for the ground-state *Z*-configurations) are in agreement with the expected reaction course. Conversely, for IIb*, the lack of an efficient overlap destabilizes this conformation and prevents the evolution toward sigma-bonded products (Figure 4, red reaction pathway and Figure 5b, red arrow). Binding through the C(4) of thiophene would require a dihydro-intermediate having an expanded valence shell for sulfur (represented in Scheme 1b by a dotted bond), a rare pattern in organosulfur chemistry that has defied every attempt we made trying to model it, as it always evolves toward the stable *Z*-double bond conformation **IIb**.

Starting from **7**, an allowed reaction pathway analogous in every way to that described above is found. The corresponding energy diagram for **7** can be found in the Supporting Information. Overall, these mechanistic scenarios corroborate the anticipated reaction pathways outlined in Figure 3 at the vertical transition stage.

### Final Photochemical Synthesis of Dithia[7]helicenes Starting from **7** and **8**

**Figure 6:**
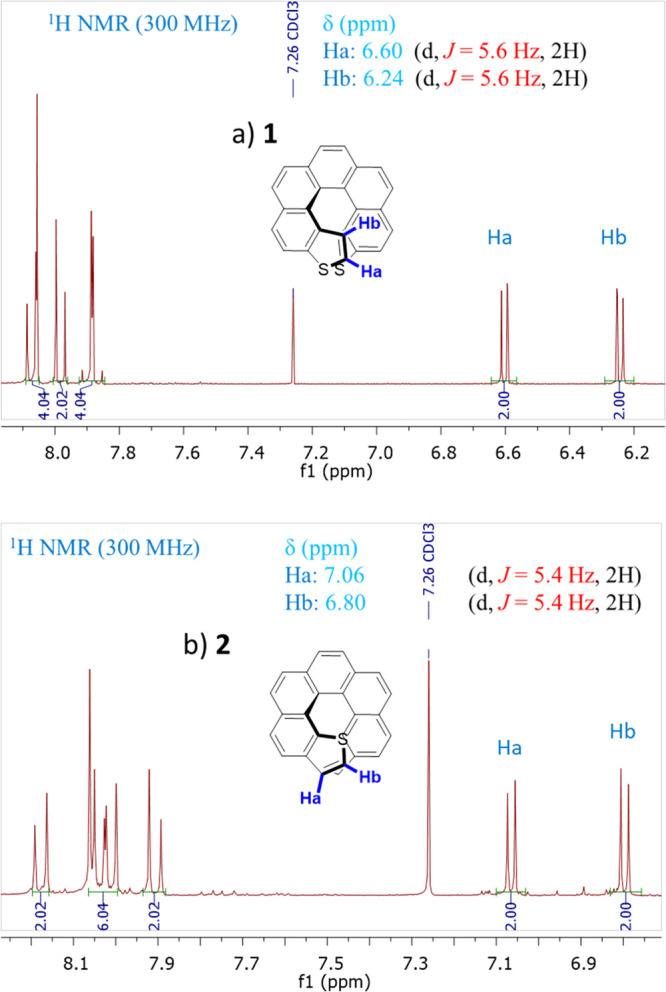
Terminal thiophene hydrogens show a distinctive pattern by ^1^H NMR when forming parts of helicenes, consisting of an upfield chemical shift and a characteristic coupling constant of these vicinal hydrogens, which makes them easily recognizable. This pattern is shown in (a) for **1** and in (b) for **2**, corroborating their naphtho[2,1-*b*]thiophene and naphtho[1,2-*b*]thiophene type of ring fusion, respectively.

**Scheme 3:**
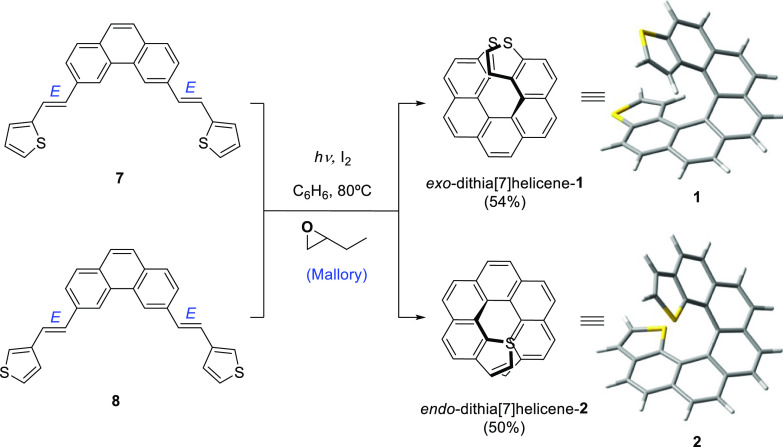
Photochemical Synthesis of Dithia[7]helicenes **1** and **2***via* a Double Photocyclization of *bis*-Stilbenic Precursors **7** and **8**

*Bis*-stilbenic compounds **7** and **8** were subjected to irradiation using a 400 W high-pressure Hg lamp to undergo a sequential double photocyclization under typical Mallory–Katz conditions^22^ (Scheme 3 and the Supporting Information). The reaction afforded the expected 3,14-dithia[7]helicene-**1** (or *exo*-dithia[7]helicene-**1**) for **7**. A distinctive fingerprint useful in the structural elucidation of this type of thiahelicenes by ^1^H NMR is the two doublets originated by the coupling of the vicinal hydrogens of the thiophene ring. They are easily spotted in the spectrum for two reasons. Even though the chemical shifts of thiophene hydrogens fall within the chemical shift of a typical aromatic hydrogen (*e.g.*, benzene), within the framework of a helicenic structure, they are shifted upfield as a consequence of the anisotropic effect of the rest of the underlying aromatic structure. Also, the coupling constant (*J*) of these vicinal hydrogens is substantially smaller and easily identified from the rest of the aromatic signals. The result is a pair of doublets in the 6–7 ppm region with *J* around 5–6 Hz. In the case of **1**, there are two doublets at 6.60 (d, *J* = 5.6 Hz, 2H) and 6.24 (dd, *J* = 5.6, 0.5 Hz, 2H), clearly identifying a naphtho[2,1-*b*]thiophene type of ring fusion (Figure 6a). For **8**, the only compound that could be isolated has two doublets at 7.06 (d, *J* = 5.4 Hz, 2H) and 6.80 (d, *J* = 5.4 Hz, 2H), consistent with a naphtho[1,2-*b*]thiophene type of ring fusion as in **2** (Figure 6b), but not with a naphtho[1,2-*c*]thiophene one, which would display a much smaller *J* (estimated *J* = 2.7–2.8 Hz from gauge-invariant atomic orbital DFT calculations, scaled using experimental *J*_s_ from Figure 6). The isolated compound was hence identified as 1,16-dithia[7]helicene-**2** (or *endo*-dithia[7]helicene-**2**). Other than the dithia[7]helicene or starting material, only intractable, unidentifiable products by NMR were obtained in different trials whether the reactions were taken to completion or not (*i.e.*, to the disappearance of the starting material). An unequivocal characterization of **1** and **2** by means of X-ray crystallography was recently reported by our group.^5^

## Conclusions

In summary, the photocyclization mechanism of 2- and 3-styrylthiophenes to afford naphtho[2,1-*b*]thiophene and naphtho[1,2-*b*]thiophene type of ring fusions, respectively, has been studied combining the conclusive information resulting from synthesis/structural elucidation with DFT and TDDFT methods to unveil mechanisms. In the first place, we carried out the syntheses of (*E*,*E*)-**7** and (*E*,*E*)-**8** and obtained their experimental UV–vis spectra. Then, we simulated these electronic spectra and analyzed the nature of their vertical electronic transitions by TDDFT. This gave us access to the excited states involved in the reaction. These excited states were geometrically optimized and analyzed again to gain information on the photochemical intermediates of the reaction. The regiochemical and stereochemical outcome of the photocyclization step is revealed at this point. Finally, back to the synthesis, the actual photocyclization of **7** and **8** afforded the theoretically anticipated dithiahelicenes **1** and **2**, the structure of which could be unambiguously assigned by ^1^H NMR spectroscopy. Full structural characterization of the resulting dithiahelicenes leaves little doubts about the findings here reported, which show the potential of excited-state quantum mechanical methods in structural and mechanistic elucidation tasks.

## Experimental Section

### General Methods

Unless otherwise stated, commercially available starting materials and solvents for chromatography and recrystallization were used without further purification. We dried and distilled over Na/K alloy tetrahydrofuran (THF), benzene, and cyclohexane immediately before using them in synthesis or photochemistry. Commercially unavailable reagents were synthetized by different means; they are explained below each one separately.

Gas chromatography analyses (GLC) were carried out with a Hewlett Packard HP-5890 instrument equipped with a flame ionization detector and a 30 m HP-5 capillary column (0.32 mm diameter and 0.25 μm film thickness) using nitrogen as carrier gas (12 psi). Column chromatography was performed with Merck silica gel 60 (0.040–0.063 μm, 240–400 mesh). Thin-layer chromatography (TLC) was performed on precoated silica gel plates (Merck 60, F254, 0.25 mm). TLC detection was done by UV_254_ light, and *R*_f_ values are given under these conditions. NMR spectra were recorded on a Bruker Avance 300 and a Bruker Avance 400 (300 and 400 MHz for ^1^H NMR and 75 and 100 MHz for ^13^C NMR, respectively, broadband proton decoupling was applied during the acquisition of ^13^C{^1^H} spectra) using CDCl_3_ as a solvent and tetramethylsilane (TMS) as an internal standard. Chemical shifts (δ) are given in ppm versus TMS. Infrared (IR) analysis was performed with a JASCO FT/IR 4100 spectrophotometer equipped with an attenuated total reflection component. Low-resolution mass spectrometry was performed using the electron impact (EI) mode at 70 eV in an AGILENT 5973N mass spectrometer coupled with an AGILENT 6890N gas chromatographer. High-resolution mass spectrometry (HRMS) analyses were carried out in an AGILENT 7200 using the EI mode at 70 eV by quadrupole time-of-flight (Q-TOF). Melting points were performed with a Reichert Thermovar polarizing light microscope and a melting point apparatus and have been corrected. A double-beam UV–vis spectrophotometer (Shimadzu UV-1603) was used for recording the electronic spectra.

### Photochemistry

A 400 W high-pressure mercury lamp (Osram HQL MBF-U) was modified by cutting away the outer glass envelope from the screw base (preserving the inner quartz arc tube containing Hg) and was mounted in a porcelain lamp holder provided with an aluminum reflector. The lamp was connected to a corresponding power unit, and the light beam was focused to a number of 100 mL Schlenck’s single-wall borosilicate tubes (0.6 mm wall thickness) placed some 10 cm away from the source, provided with magnetic stirring and a vertical condenser refrigerated with a recirculating chiller (Huber MPC-K6) using a 30% ethylene glycol–water mixture as a coolant. The chemical hood was lined with aluminum foil to avoid unwanted exposure to UV radiation. We used cyclohexane or benzene under reflux as solvents.

### Synthesis and Characterization of Compounds

Experimental Procedures for the Synthesis of Intermediates and Final Products

#### (*E*)-1,2-bis(4-Bromophenyl)ethene (**3**)^23^

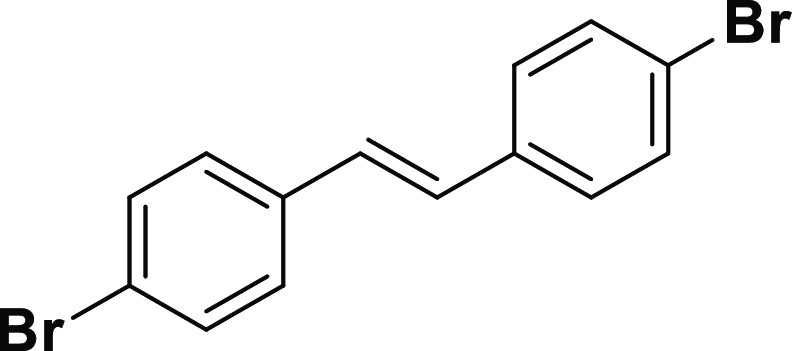



This compound was prepared by the procedure described in the literature.^23^ In a 100 mL round flask, 4-bromobenzyl bromide (5.00 g, 20 mmol, 2 equiv), sodium *p*-toluenesulfinate (1.78 g, 10 mmol, 1 equiv), and KOH (1.68 g, 30 mmol, 3 equiv) were added, and then 25 mL of dimethyl sulfoxide (DMSO) was added. The mixture was heated in an oil bath under reflux (100 °C) for 24 h. After cooling to room temperature, the solvent (DMSO) was removed by vacuum distillation, and a brown solid was obtained. The product was purified by column chromatography on silica gel (hexane) to afford a white solid (2.772 g, 82% yield). A different way to purify the product was by recrystallization from EtOH/CHCl_3_ (3:1), obtaining **3** as white crystals.

White solid; mp 195.3 °C (corrected); *R*_f_ = 0.53 (hexane); ^1^H NMR (CDCl_3_, 300 MHz): δ = 7.51–7.46 (m, 4H), 7.39–7.34 (m, 4H), and 7.02 (s, 2H). ^13^C{^1^H} NMR (CDCl_3_, 75 MHz): δ = 136.1, 132.0, 128.3, 128.2, and 121.8. MS (EI) *m/z*: 339.90 (M^+^ + 4, 31.7), 337.90 (M^+^ + 2, 61.2), 335.90 (M^+^, 32.5), 281.05 (22.2), 258.00 (6.8), 207.05 (53.4), 180.00 (2.7), 179.10 (17.9), 178.10 (100), 176.10 (24.7), 152.15 (10.3), 126.05 (3.3), 89.05 (23.0), and 88.10 (21.0). IR (neat) ν_max_: 3020, 2924, 1581, 1481, 1404, 1323, 1215, 1068, 999, 968, 822, and 710 cm^–1^.

#### 3,6-Dibromophenanthrene (**4**)^24^

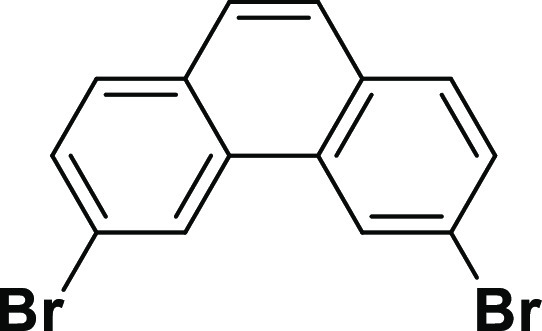



This compound was prepared using the main photochemical setup described above. A solution/suspension of (*E*)-1,2-bis(4-bromophenyl)ethene (33.6 mg, 0.1 mmol; 1 equiv) and KI (16.6 mg, 0.1 mmol; 1 equiv) in cyclohexane (100 mL) was prepared in a Schlenk tube provided with a vertical condenser open to the air connected to a chiller. The recirculating chiller was turned on, and the mixture was irradiated with a 400 W high-pressure Hg lamp for 3–4 h under reflux. The progress of the reaction was monitored by GLC. After the reaction was completed, the crude mixture was washed with NaHSO_3_, dried under magnesium sulfate, and filtered, and the solvent was evaporated under reduced pressure (15 Torr). The product was purified by column chromatography of silica gel (hexane) to afford a white solid (26.8 mg, 80% purified yield).

White solid; mp 191.6 °C (corrected); *R*_f_ = 0.53 (hexane); ^1^H NMR (CDCl_3_, 300 MHz): δ = 8.72 (d, *J* = 1.7 Hz, 2H), 7.76 (d, *J* = 8.3 Hz, 2H), and 7.73–7.69 (m, 4H). ^13^C{^1^H} NMR (CDCl_3_, 75 MHz): δ = 131.0, 130.9, 130.6, 130.2, 126.9, 125.7, and 121.4. MS (EI) *m/z*: 337.90 (M^+^ + 4, 50.0), 335.90 (M^+^ + 2, 100), 333.90 (M^+^, 50.2), 176.10 (8.1), 150.10 (10), and 88.05 (29). IR (neat) ν_max_: 2923, 2851, 1901, 1586, 1494, 1429, 1407, 1381, 1153, 1106, 1069, 1017, 856, 846, 835, and 769 cm^–1^.

#### (*E*)-4,4,5,5-Tetramethyl-2-(2-(thiophen-2-yl)vinyl)-1,3,2-dioxoborolane (**5**)^25^

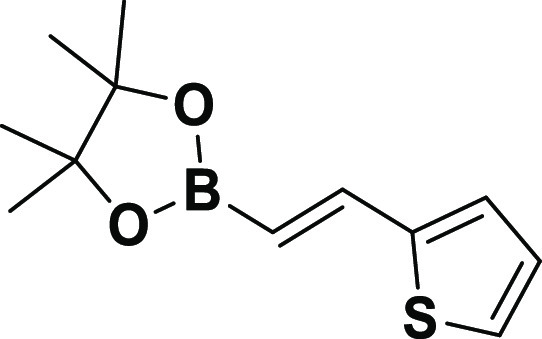



This compound was prepared by adapting a procedure from the literature.^18^ In an oven-dried Schlenk tube CuCl (2.96 mg; 0.03 mmol; 0.06 equiv), NaO*t*-Bu (5.76 mg; 0.06 mmol; 0.12 equiv) and xantphos ligand (17.35 mg; 0.03 mmol; 0.06 equiv) were added, and the reaction mixture was then subjected to three cycles of vacuum/argon. Then, 1 mL of dry THF was injected, and the solution was stirred for 30 min at room temperature. Next, bis(pinacolato)diboron (253.94 mg; 1 mmol; 2 equiv) in 0.5 mL of dry THF was added. The solution was stirred for 10 min at room temperature, and 2-ethynylthiophene (54.08 mg; 0.5 mmol; 1 equiv) was added, followed by MeOH (42 μL, 1 mmol). The reaction mixture was stirred at room temperature overnight (no starting material was detected by TLC). Then, it was filtered through a pad of celite, and the residue was purified by preparative TLC (silica gel, hexane–EtOAc 9:1) obtaining a pale yellow oil (47.2 mg, 40% yield).

Pale yellow oil; *R*_f_ = 0.51 (hexane–EtOAc 9:1); ^1^H NMR (CDCl_3_, 300 MHz): δ = 7.47 (d, *J* = 18.1 Hz, 1H), 7.24 (d, *J* = 5.1 Hz, 1H), 7.08 (d, *J* = 3.5 Hz, 1H), 6.99 (dd, *J* = 5.0 3.6 Hz, 1H), 5.91 (d, *J* = 18.1 Hz, 1H), and 1.30 (s, 12H). ^13^C{^1^H} NMR (CDCl_3_, 75 MHz): δ = 144.1, 141.9, 127.8, 127.8, 126.4, 83.5, and 24.9. MS (EI) *m/z*: 238.1 (M^+^ + 2, 5.6), 237.1 (M^+^ + 1, 13.4), 236.1 (M^+^, 88.8), 235 (M^+^ – 1, 23.2), 221.1 (20.5), 193.05 (7.3), 178.05 (10.7), 163.00 (26.8), 151.10 (41.1), 136.00 (100), 111.00 (27.9), 85.10 (8.9), and 57.10 (5.2). IR (neat) ν_max_: 2974, 2911, 2168, 1616, 1520, 1458, 1423, 1373, 1327, 1234, 1146, 976, 910, 849, and 733 cm^–1^.

#### (*E*)-4,4,5,5-Tetramethyl-2-(2-(thiophen-3-yl)vinyl)-1,3,2-dioxoborolane (**6**)^25^

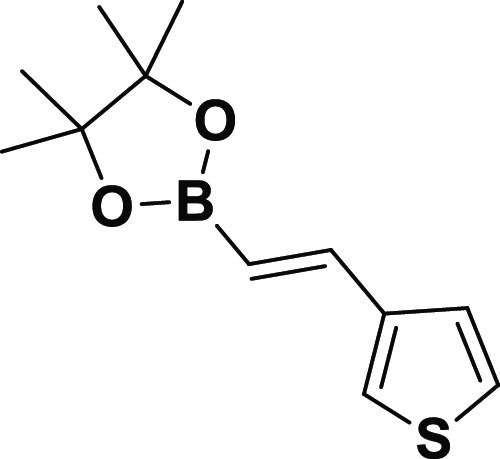



This compound was prepared following the previous procedure, replacing the terminal alkyne by 3-ethynylthiophene as a pale yellow oil (54.3 mg, 46% yield).

Pale yellow oil; *R*_f_ = 0.52 (hexane–EtOAc 9:1); ^1^H NMR (CDCl_3_, 300 MHz): δ = 7.38 (d, *J* = 18.4 Hz, 1H), 7.32–7.28 (m, 2H), 7.28–7.24 (m, 1H), 5.95 (d, *J* = 18.3 Hz, 1H), and 1.30 (s, 12H). ^13^C{^1^H} NMR (CDCl_3_, 75 MHz): δ = 143.3, 141.3, 126.2, 125.1, 125.0, 83.4, and 24.9. MS (EI) *m/z*: 238.10 (M^+^ + 2, 4.1), 237.10 (M^+^ + 1, 10.4), 236.10 (M^+^, 70.2), 235.10 (M^+^ – 1, 15.6), 221.10 (17.5), 192.10 (7.2), 178.10 (26.5), 163.05 (54.8), 150.10 (26.2), 136.00 (100), 110.05 (12.1), 85.05 (9.4), and 57.10 (5.5). IR (neat) ν_max_: 2927, 2858, 2167, 2025, 1724, 1624, 1516, 1458, 1331, 1261, 1146, 991, 849, 771, and 690 cm^–1^.

#### 3,6-bis((*E*)-2-(Thiophen-2-yl)vinyl)phenanthrene (**7**)

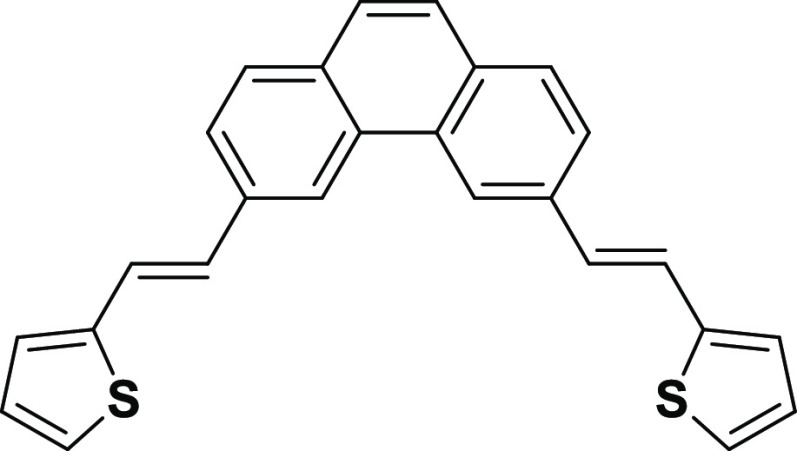



This compound was prepared by adapting to our substrates a Suzuki coupling described in the literature.^26^ In an oven-dried pressure tube, PdCl_2_ (17.73 mg; 0.1 mmol; 0.20 equiv), PPh_3_ (52.45 mg; 0.2 mmol; 0.40 equiv), Cs_2_CO_3_ (977.46 mg; 3 mmol; 6 equiv), and 3,6-bibromophenanthrene (168.3 mg; 0.5 mmol; 1 equiv) were added. The tube was sealed with a septum, and after being subjected to three cycles of vacuum/argon, 4,4,5,5-tetramethyl-2-(2-(thiophen-2-yl)vinyl)-1,3,2-dioxoborolane (360.3 mg; 1.5 mmol; 3 equiv) dissolved in 3.6 mL of THF was added with a syringe, followed by 0.4 mL of H_2_O. The threaded tube was closed and heated in an oil bath at 85 °C for 20–24 h. The reaction was monitored by TLC. After the reaction was completed, it was extracted using 10 mL of H_2_O and 3 × 10 mL of CH_2_Cl_2_. A rather insoluble solid in suspension was observed in the organic phase. The product, yellow solid, was separated by filtration. On the other hand, the combined extracts were solvent-evaporated and purified by column chromatography with silica gel (hexane–EtOAc 9:1) to obtain an additional fraction of the same yellow solid which was incorporated to the formerly filtered solid. The product was obtained as a yellow solid (122.3 mg, 62% yield).

Yellow solid; mp 203.8 °C (corrected); *R*_f_ = 0.55 (hexane–EtOAc 9:1); ^1^H NMR (CDCl_3_, 400 MHz): δ = 8.66 (d, *J* = 0.65 Hz, 2H), 7.84 (d, *J* = 8.3 Hz, 2H), 7.77 (dd, *J* = 8.4, 1.4 Hz, 2H), 7.67 (s, 2H), 7.46 (d, *J* = 16.0 Hz, 2H), 7.26 (d, *J* = 3.5 Hz, 2H), 7.23 (d, *J* = 16.0 Hz, 2H) 7.17 (d, *J* = 3.5 Hz, 2H), and 7.06 (dd, *J* = 5.1, 3.5 Hz, 2H).^27^ MS (EI, DIP) *m/z*: 396.2 (M^+^ + 2, 24.7), 395.2 (M^+^ + 1, 58.6), 394.2 (M^+^, 100), 359.1 (7.1), 308.1 (10.3), 276.1 (8.7), 197.1 (18.8), 179.3 (5.8), and 154.2 (3.4). IR (neat) ν_max_: 3097, 1786, 1608, 1508, 1423, 1342, 1192, 949, 887, 845, and 698 cm^–1^. HRMS (EI/Q-TOF) *m/z*: [M]^+^ calcd for C_26_H_18_S_2_, 394.0850; found, 394.0853.

#### 3,6-bis-((*E*)-2-(Thiophen-3-yl)vinyl)phenanthrene (**8**)

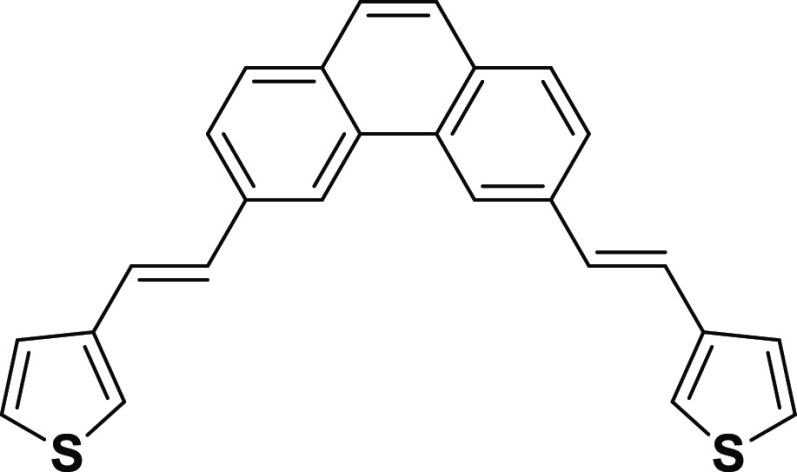



This compound was prepared following the previous procedure but using 4,4,5,5-tetramethyl-2-(2-(thiophen-3-yl)vinyl)-1,3,2-dioxoborolane as the starting reagent. The product was obtained as a yellow solid (146.0 mg, 74% yield).

Yellow solid in a 74% yield; mp 193.4 °C (corrected); *R*_f_ = 0.60 (hexane–EtOAc 9:1); ^1^H NMR (CDCl_3_, 400 MHz): δ = 8.66 (s, 2H), 7.84–7.75 (m, 4H), 7.65 (s, 2H), 7.45 (d, *J* = 4.2 Hz, 2H), 7.40–7.29 (m, 6H), and 7.23 (d, *J* = 16.0 Hz, 2H).^27^ MS (EI, DIP) *m/z* 396.1 (M^+^ + 2, 13.0), 395.1 (M^+^ + 1, 30.3), 394.1 (M^+^, 100), 360.1 (9.8), 309.1 (14.1), 284.1 (9.4), and 197.0 (9.1). IR (neat) ν_max_: 3089, 1608, 1408, 1203, 1087, 957, 845, 771, and 690 cm^–1^. HRMS (EI/Q-TOF) *m/z*: [M]^+^ calcd for C_26_H_18_S_2_, 394.0850; found, 394.0848.

#### 3,14-Dithia[7]helicene (**1**)^5,14^

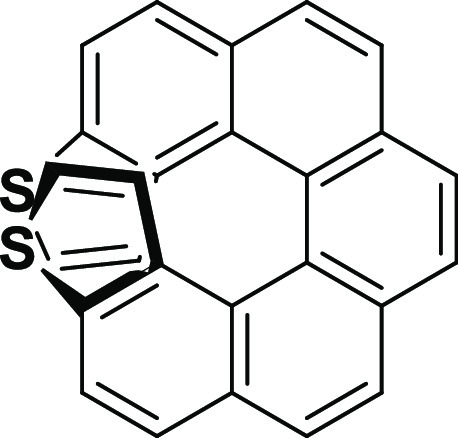



The reaction was run in parallel in two oven-dried Schlenk tubes, loaded with a suspension of 3,6-bis((*E*)-2-(thiophen-2-yl)vinyl)phenanthrene (19.73 mg; 0.05 mmol; 1 equiv), I_2_ (38.07 mg; 0.15 mmol; 3 equiv), and 1,2-epoxybutane (360.50 mg; 5 mmol; 100 equiv) in 100 mL of benzene each. The tubes were provided with vertical condensers connected to a chiller, and Ar was bubbled into the solution during the reaction. The recirculation chiller was turned on, and the mixture was irradiated with a 400 W high-pressure Hg lamp for 3 h under reflux. The progress of the reaction was monitored by TLC. After the reaction was completed, it was washed with aqueous NaHSO_3_, dried over magnesium sulfate, and filtered, and the solvent was evaporated under a reduced pressure (15 Torr). The residue was purified by column chromatography on silica gel (hexane–CH_2_Cl_2_ 8:2) to obtain a yellow solid. A 54% yield was calculated by ^1^H NMR using 1,3,5-trimethoxybenzene (99%) as the internal standard. Recrystallization from EtOAc afforded slightly brown, near colorless crystals (6.8 mg, 35% yield).

Light brown, near colorless crystals; *R*_f_ = 0.48 (hexane/AcOEt 9:1); ^1^H NMR (CDCl_3_, 300 MHz): δ = 8.07 (d, *J* = 8.9 Hz, 2H), 8.06 (s, 2H), 7.98 (d, *J* = 8.4 Hz, 2H), 7.93–7.84 (m, 4H), 6.60 (d, *J* = 5.6 Hz, 2H), and 6.24 (d, *J* = 5.6, 2H). ^13^C{^1^H} NMR (CDCl_3_, 75 MHz): δ = 138.6, 135.3, 132.2, 129.9, 127.9, 127.9, 127.1, 125.4, 125.1, 124.6, 124.2, 123.0, and 121.4.

#### 1,16-Dithia[7]helicene (**2**)^5^

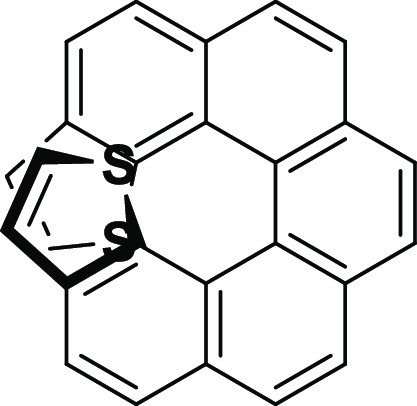



In this case, a suspension of 3,6-bis((*E*)-2-(thiophen-3-yl)vinyl)phenanthrene (19.72 mg; 0.05 mmol; 1 equiv), I_2_ (38.07 mg; 0.15 mmol, 3 equiv), and 1,2-epoxybutane (360.50 mg; 5 mmol; 100 equiv) in benzene was irradiated following the same overall methodology described above for the *exo* isomer. A 50% yield was calculated by ^1^H NMR using 1,3,5-trimethoxybenzene (99%) as the internal standard. Recrystallization from EtOAc afforded yellow crystals (5.8 mg, 30% yield).

Yellow crystals; *R*_f_ = 0.35 (hexane/AcOEt 9:1); ^1^H NMR (CDCl_3_, 300 MHz): δ = 8.18 (d, *J* = 8.4 Hz, 2H), 8.07–7.99 (m, 6H), 7.91 (d, *J* = 8.4 Hz, 2H), 7.06 (d, *J* = 5.4 Hz, 2H), and 6.80 (d, *J* = 5.4 Hz, 2H). ^13^C{^1^H} NMR (CDCl_3_, 75 MHz): δ = 138.2, 136.5, 132.8, 130.6, 128.8, 128.3, 126.8, 125.6, 125.2, 124.1, 123.1, 122.8, and 122.6.

### Calculation Details

For the UV–vis spectral simulation, we performed a series of DFT geometry optimizations to understand the conformational equilibria of **7** and **8**, followed by TDDFT on the major conformers in order to find the most appropriate functional for this job. Our experimental UV–vis spectra were used as a benchmark in all cases for spectral fitting. In general, all the methods tested provided comparable results and a reasonable fit, indicating that this is not an intrinsically difficult spectral simulation. The double hybrid functional with a dispersion correction term wB97X-D,^28^ in *n*-hexane as the PCM, was finally chosen for both DFT and TDDFT calculations. Pople’s split-valence quasi triple-ζ, in the valence shell basis set and addition of both polarization and diffuse functions, 6-311++G(d,p) was used in the ground state for geometry optimization and vibrational analysis, while a more expanded basis set, 6-311++G(2df,2pd), was used for UV–vis spectral calculations.^29^ These calculations were performed using Gaussian09 suite of programs.^30^ For the excited states, optimization and vibrational analysis were carried out with Gaussian16,^31^ which provides the tools to perform analytical vibrational analysis to TDDFT-optimized excited states.

## Supporting Information Available

^1^H (**1–8**) and ^13^C{^1^H} NMR (**1–6**) spectra, melting point corrections applied and UV–vis spectra (**7**,**8**) of these compounds, calculation details on the simulation of the UV–vis spectra, conformational analysis, NTOs of the relevant UV–vis bands, energies of the optimized excited states, and NTO analysis (PDF)
