# Supplementary material for: Exploring the Photocyclization Pathways of Styrylthiophenes in the Synthesis of Thiahelicenes: When the Theory and Experiment Meet
Source: J Org Chem. 2021 Mar 26;86(8):5668–79. doi: 10.1021/acs.joc.1c00147 (PMC8459451; doi:10.1021/acs.joc.1c00147)
Supplement: Supplementary file 1 — jo1c00147_si_001.pdf [file jo1c00147_si_001.pdf]

Supporting Information

Exploring the Photocyclization Pathways of Styrylthiophenes in the Synthesis of Thiahelicenes: When Theory and Experiment Meet

Bianca C. Baciu,[1] José Antonio Vergés,[2] and Albert Guijarro,[1]*

[1]Departamento de Química Orgánica and Instituto Universitario de Síntesis Orgánica
Campus de San Vicente del Raspeig, Universidad de Alicante, Apdo. 99, 03080 Alicante, Spain.
[2]Departamento de Teoría y Simulación de Materiales,
Instituto de Ciencia de Materiales de Madrid (CSIC), Cantoblanco, 28049, Madrid, Spain

E-mail: aguijarro@ua.es

Table of Contents

1. ^{1}H NMR and ^{13}C NMR spectra of compounds S3

1.1. (*E*)-1,2-bis(4-bromophenyl)ethene (300MHz, CDCl3) S3

1.2. 3,6-dibromophenanthrene (300MHz, CDCl3) S4

1.3. 4,4,5,5-tetramethyl-2-(2-(thiophen-2-yl)vinyl)-1,3,2-dioxoborolane (300MHz, CDCl3) S5

1.4. 4,4,5,5-tetramethyl-2-(2-(thiophen-3-yl)vinyl)-1,3,2-dioxoborolane (300MHz, CDCl3) S6

1.5. 3,6-bis-((*E*)-2-(thiophen-2-yl)vinyl)phenanthrene (400MHz, CDCl3) S7

1.6. 3,6-bis((E)-2-(thiophen-3-yl)vinyl)phenanthrene (400MHz, CDCl3) S7

1.7. *Exo*-dithia[7]helicene (300MHz, $CDCl_3$) S8

1.8. *Endo*-dithia[7]helicene (300MHz, $CDCl_3$) S9

2. Melting point correction S10

3. Experimental UV-vis spectra of 7 and 8 .... S11

4. DFT and TDDFT Calculations S13

4.1. Conformational analysis of **7** and **8** S13

4.2. Natural Transition Orbitals (NTOs) of the relevant UV-vis bands in **7** and **8** S16

4.3. Diagram and Table of energies of the optimized geometries of ground and excited states (*) of **7**, calculated at the WB97XD/6-311++G(2d,2p) level in n-hexane as PCM. S20

4.4. Optimized geometries of excited states of **7** in S_1**: I***, **II*** S20

4.5. Diagram and Table of energies of the optimized geometries of ground and excited states (*) of **8**, calculated at the WB97XD/6-311++G(2d,2p) level in n-hexane as PCM. S23

4.6. Optimized geometries of excited states of **8** in S_1**: I***, **IIa***, **IIb*** S23

4.7. Natural Transition Orbitals (NTOs) of the geometrically optimized S_1 excited state of **7** S27

4.8. Natural Transition Orbitals (NTOs) of the geometrically optimized S_1 excited state of **8** S27

1. ^{1}H NMR and ^{13}C NMR spectra of compounds

1.1. (*E*)-1,2-bis(4-bromophenyl)ethane (**3**) (300MHz, CDCl3)

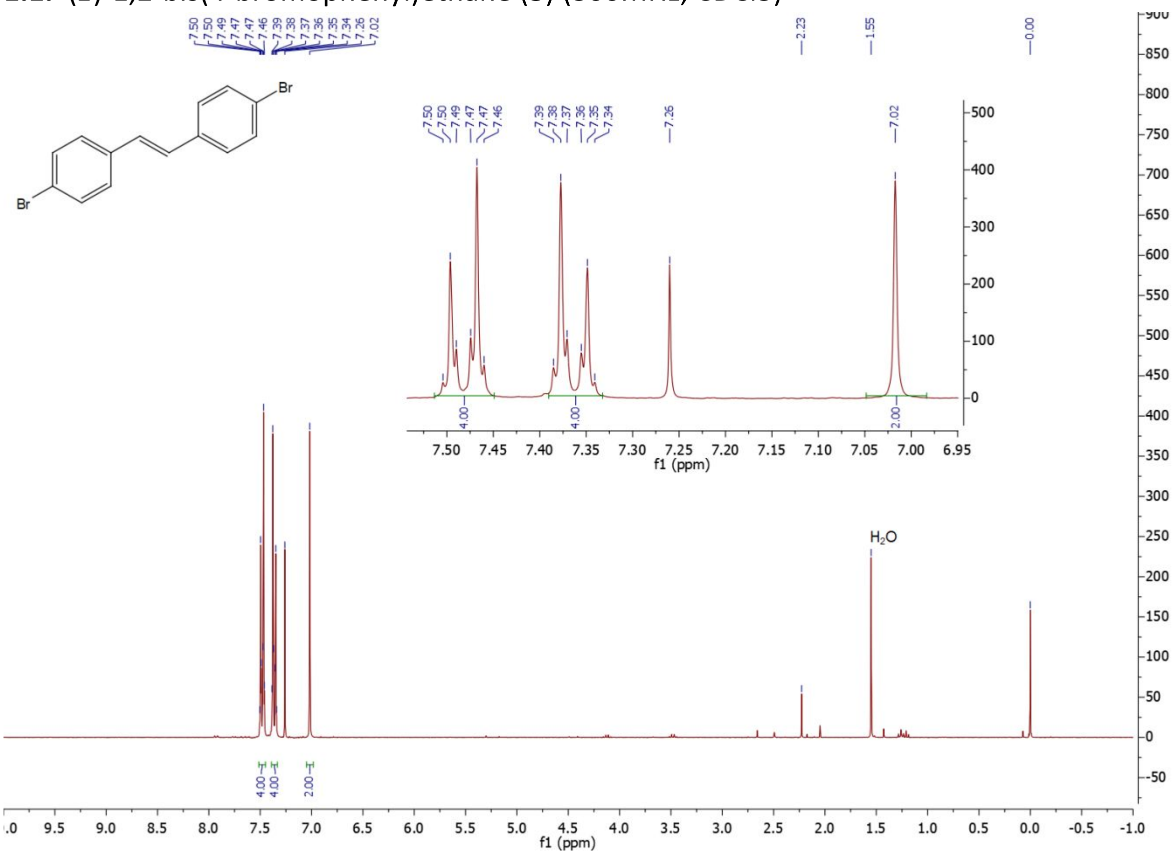

(*E*)-1,2-bis(4-bromophenyl)ethane (**3**) (75MHz, $CDCl_3$)

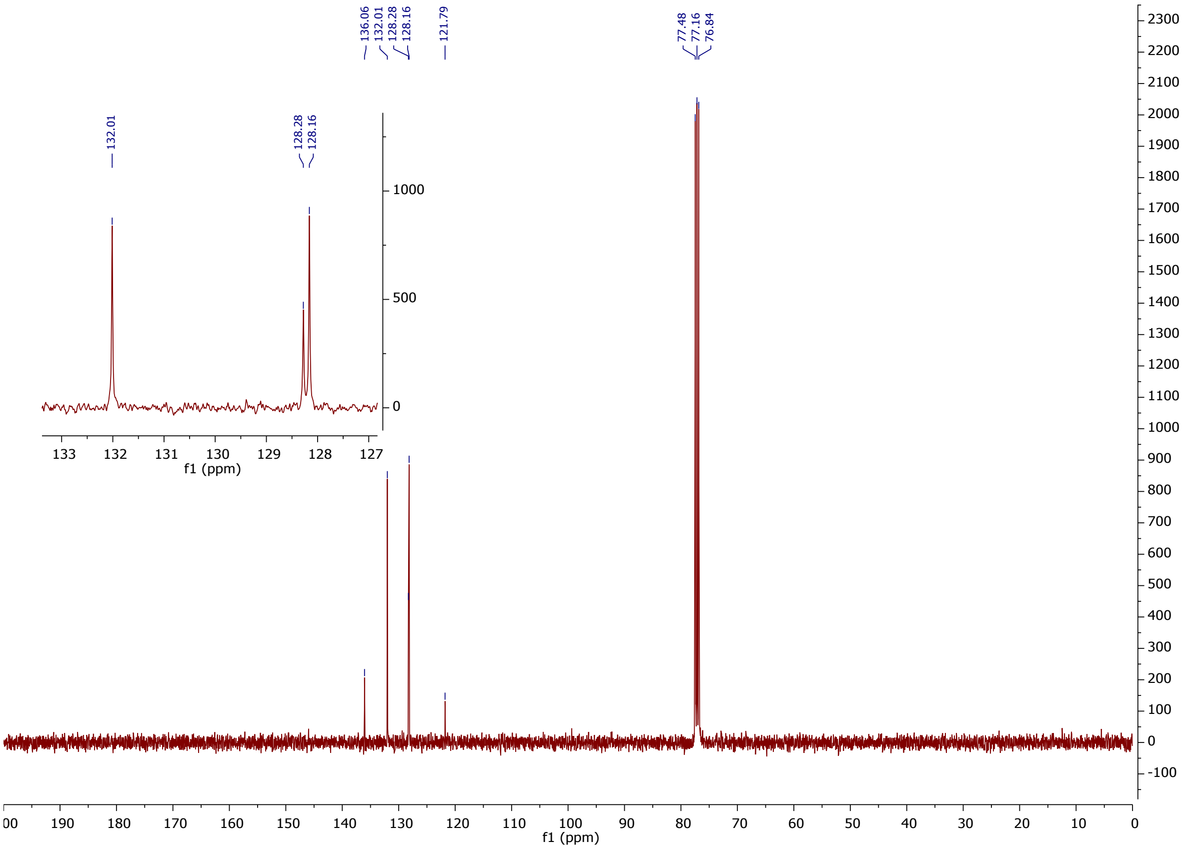

1.2. 3,6-dibromophenanthrene (**4**) (300MHz, CDCl3)

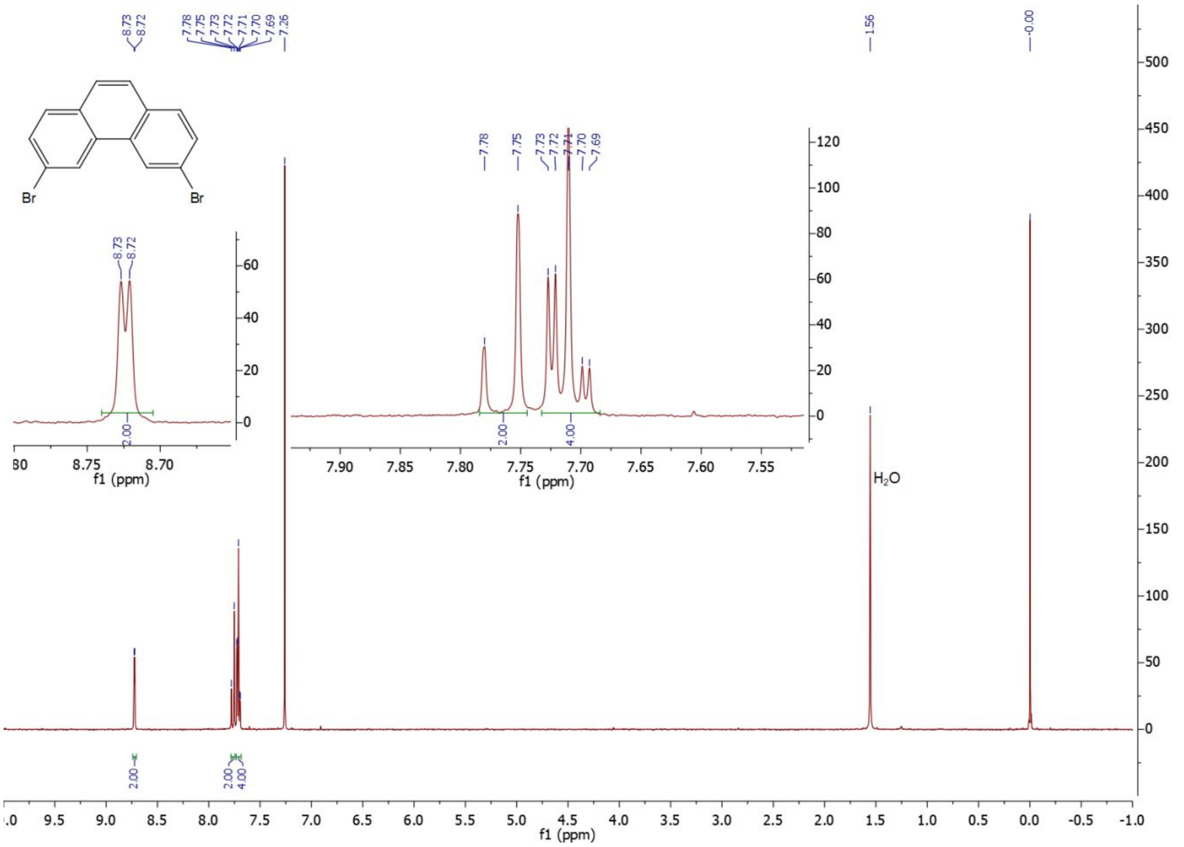

3,6-dibromophenanthrene (**4**) (75MHz, $CDCl_3$)

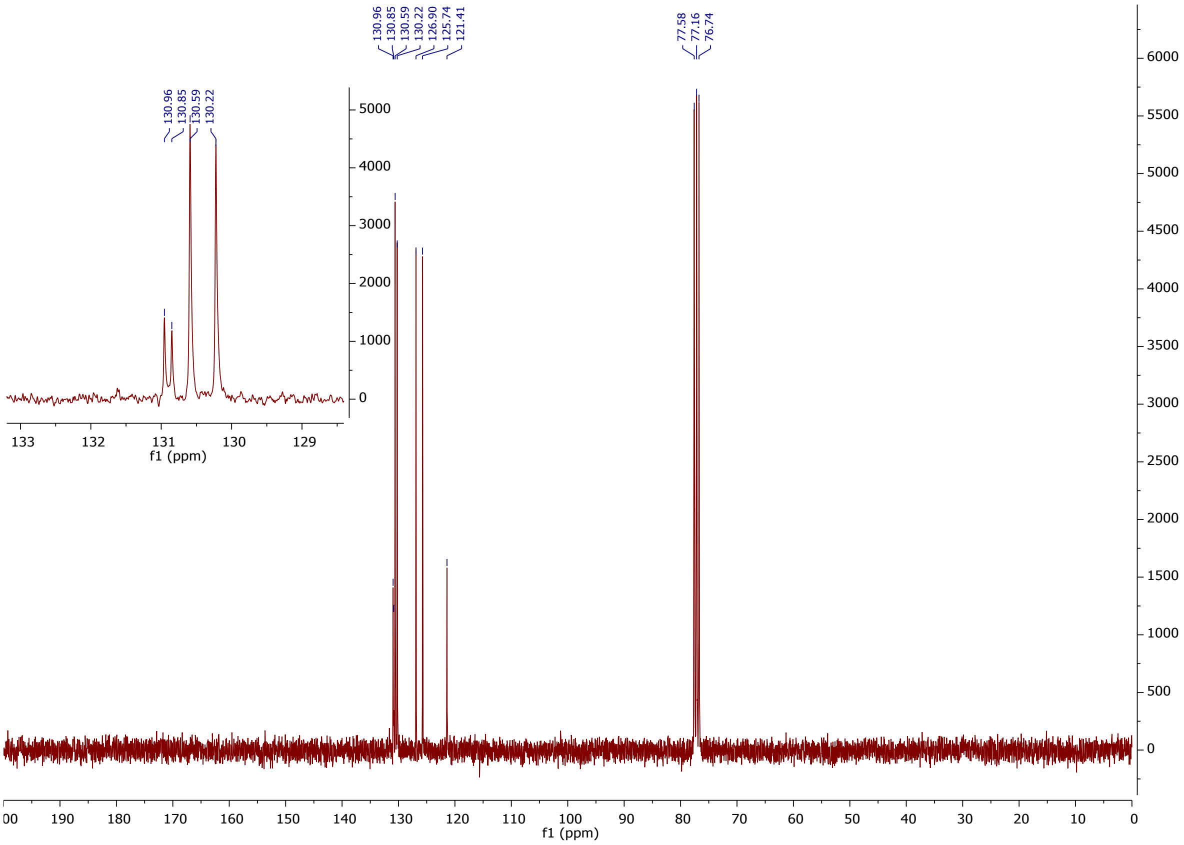

1.3. 4,4,5,5-tetramethyl-2-(2-(thiophen-2-yl)vinyl)-1,3,2-dioxoborolane (**5**) (300MHz, CDCl3)

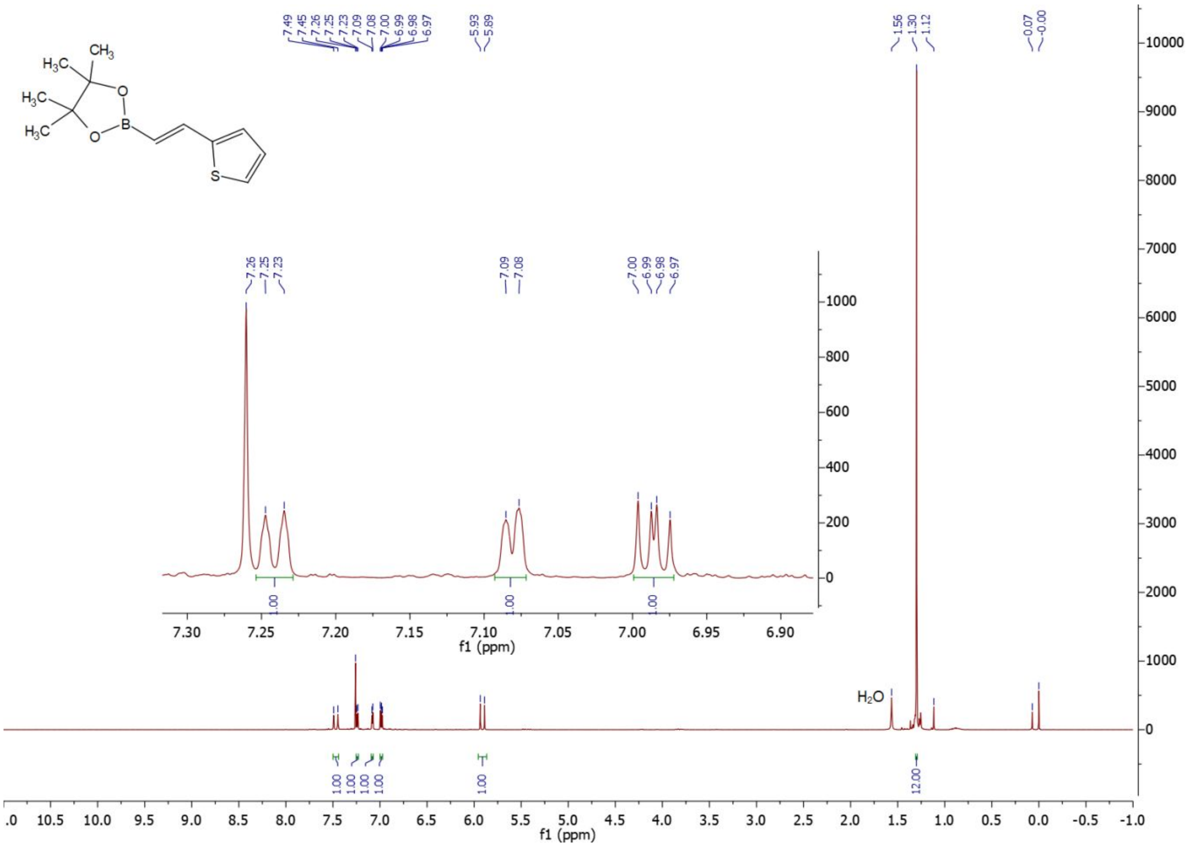

4,4,5,5-tetramethyl-2-(2-(thiophen-2-yl)vinyl)-1,3,2-dioxoborolane (**5**) (75MHz, $CDCl_3$)

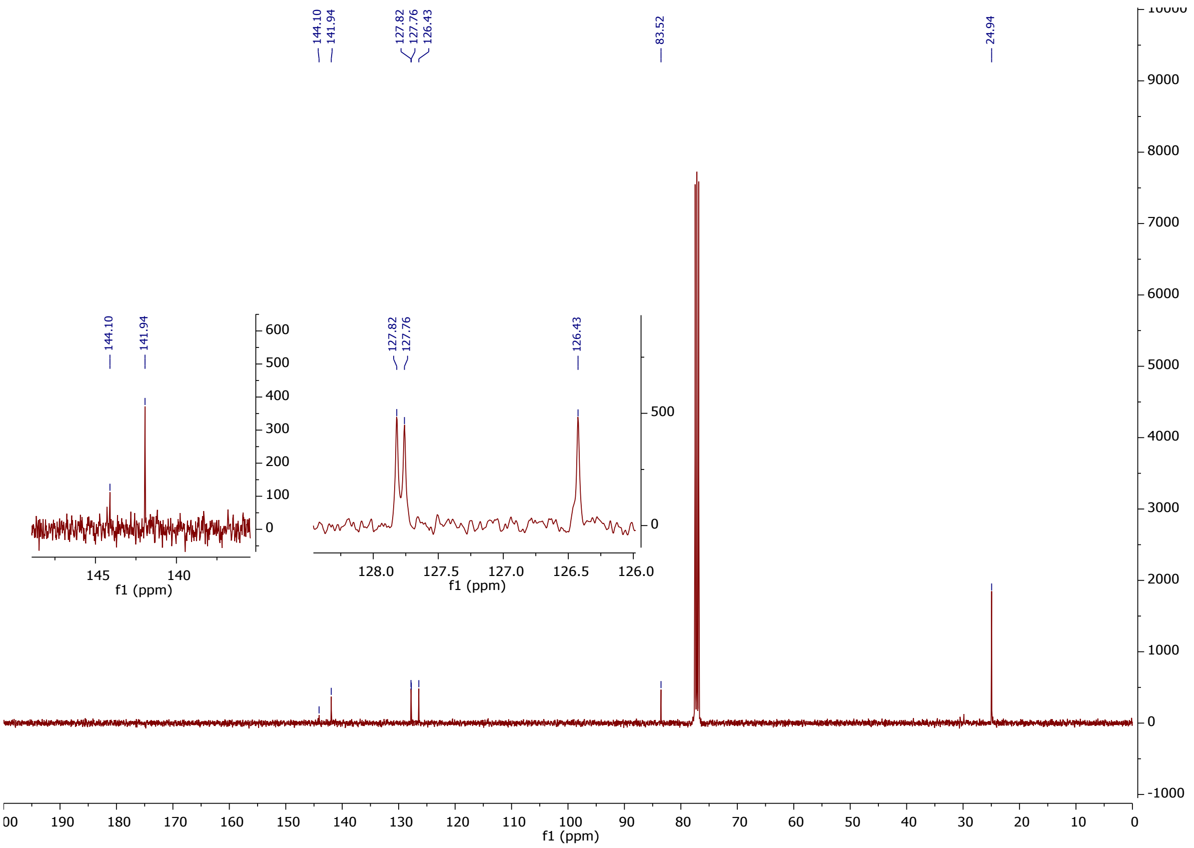

1.4. 4,4,5,5-tetramethyl-2-(2-(thiophen-3-yl)vinyl)-1,3,2-dioxoborolane (**6**) (300MHz, CDCl3)

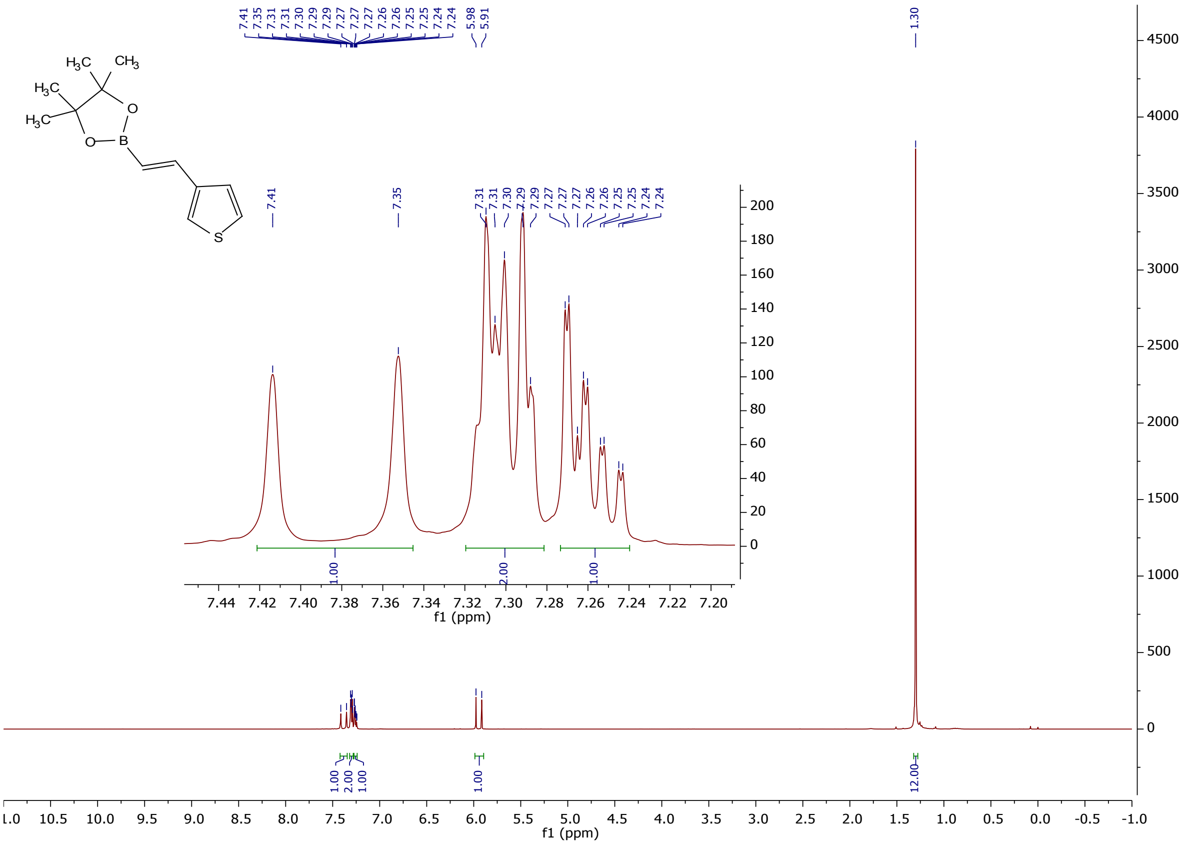

4,4,5,5-tetramethyl-2-(2-(thiophen-2-yl)vinyl)-1,3,2-dioxoborolane (**6**) (75MHz, $CDCl_3$)

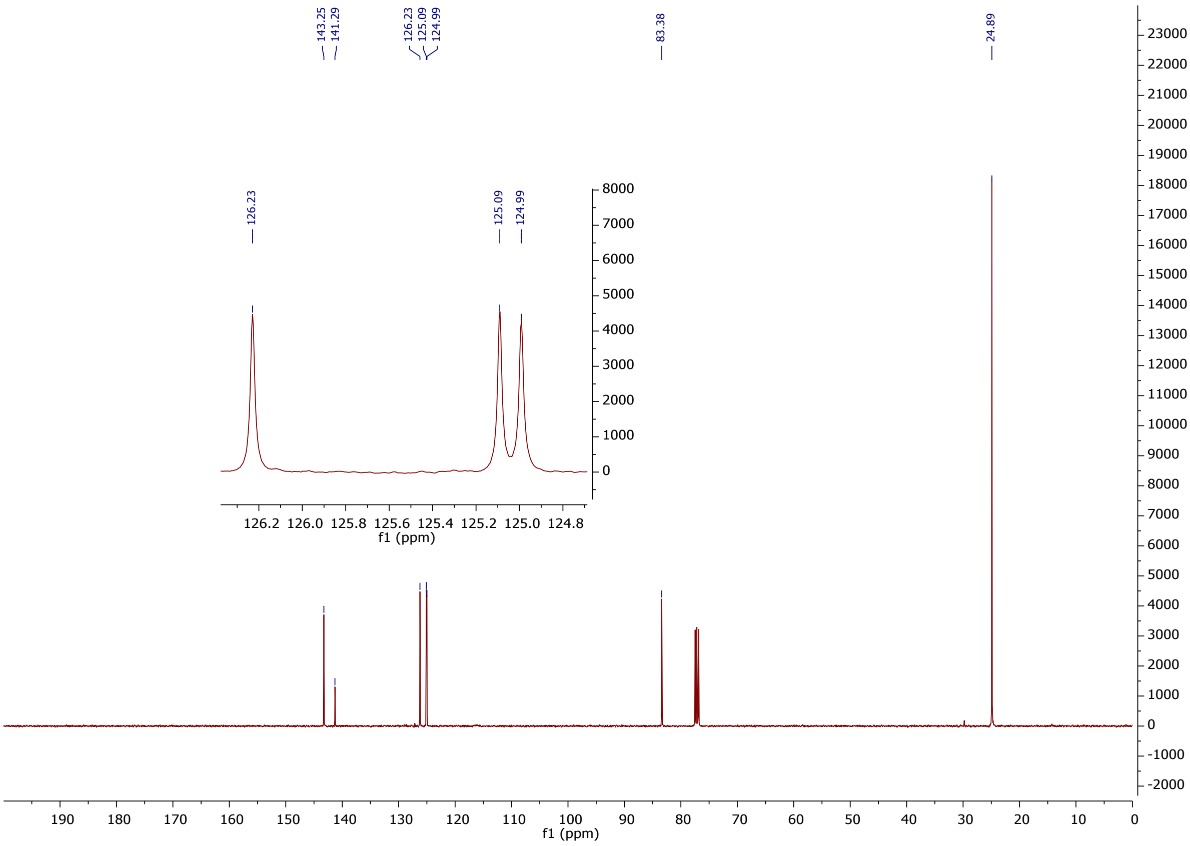

1.5. 3,6-bis-((*E*)-2-(thiophen-2-yl)vinyl)phenanthrene (**7**) (400MHz, CDCl3)

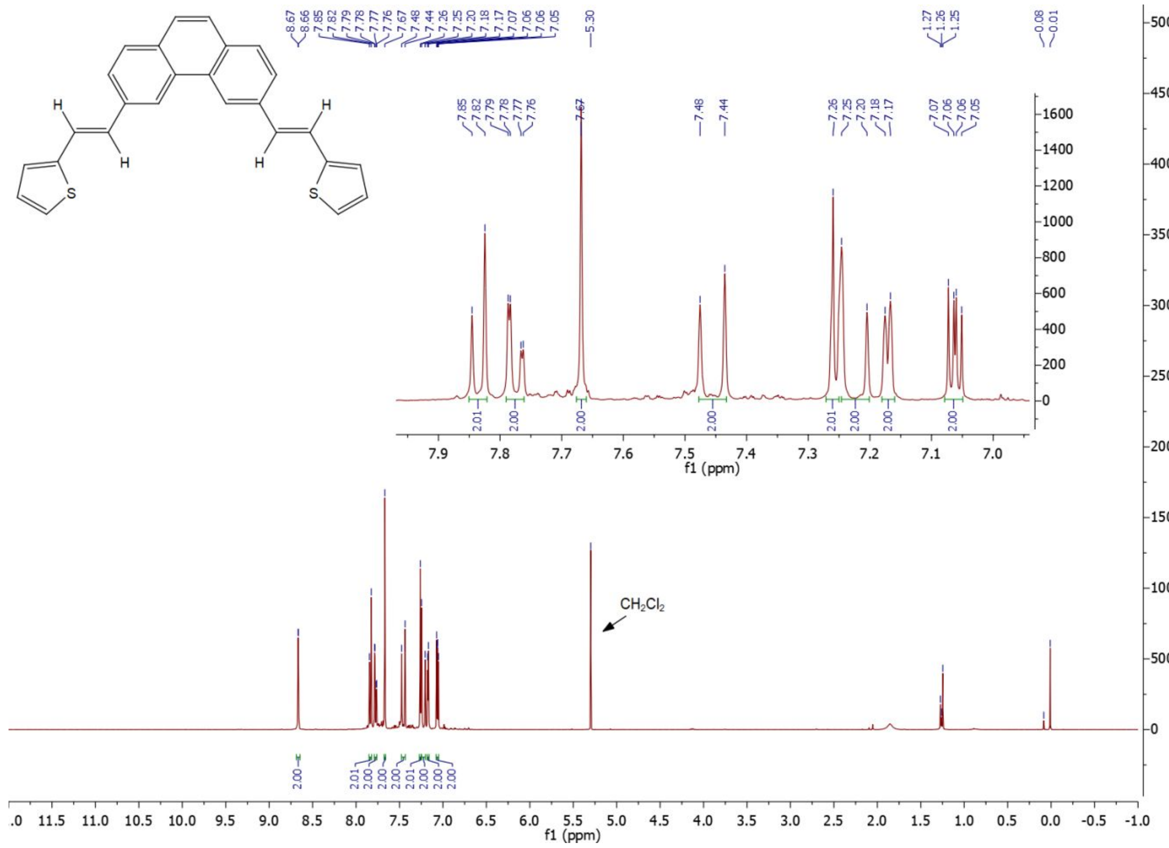

1.6. 3,6-bis((E)-2-(thiophen-3-yl)vinyl)phenanthrene (**8**) (400MHz, CDCl3)

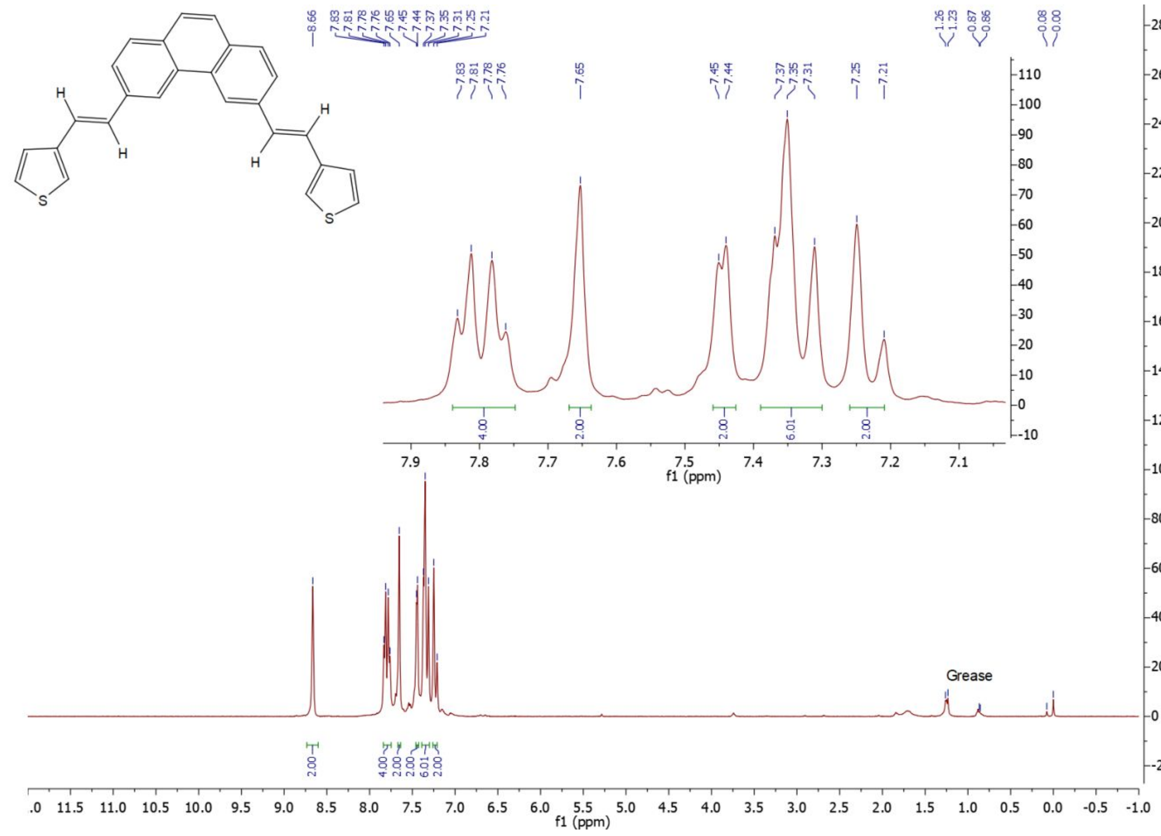

1.7. *Exo*-dithia[7]helicene (**1**) (300MHz, $CDCl_3$)

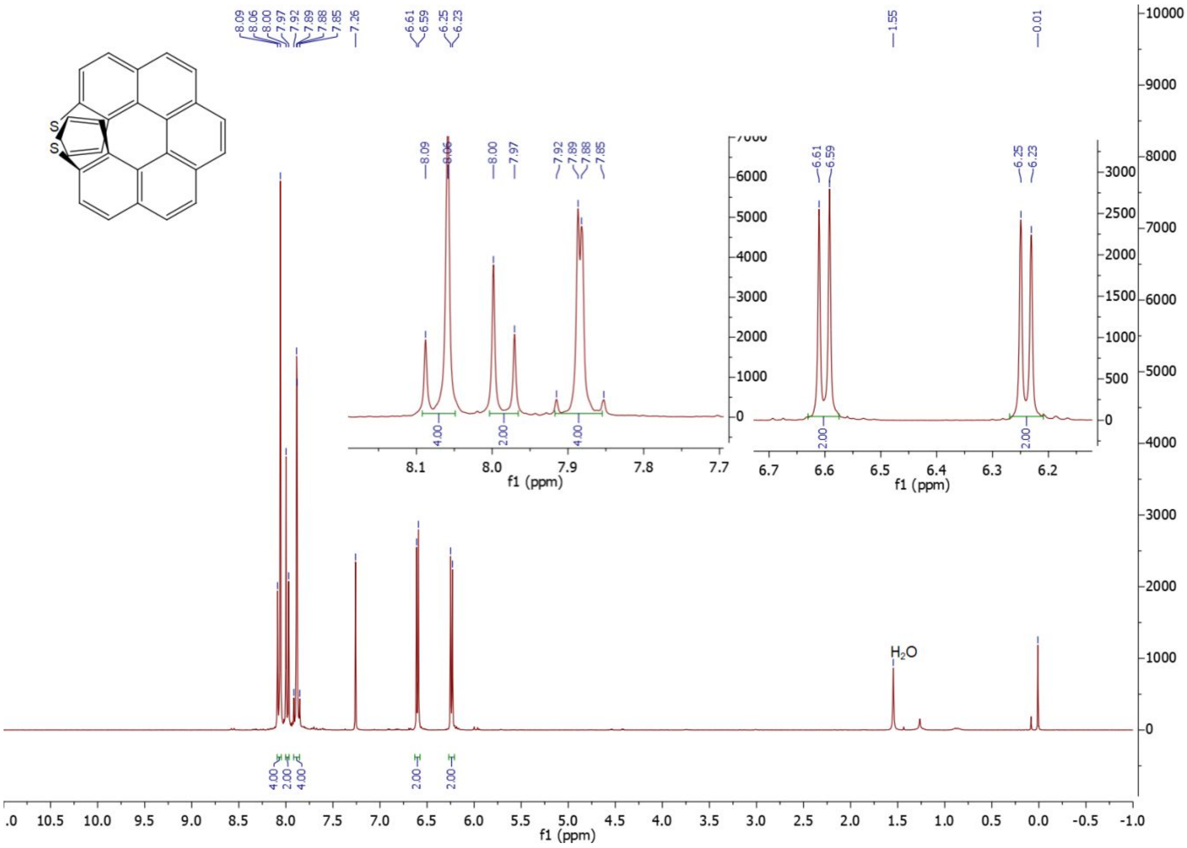

Exo-dithia[7]helicene (**1**) (75MHz, $CDCl_3$)

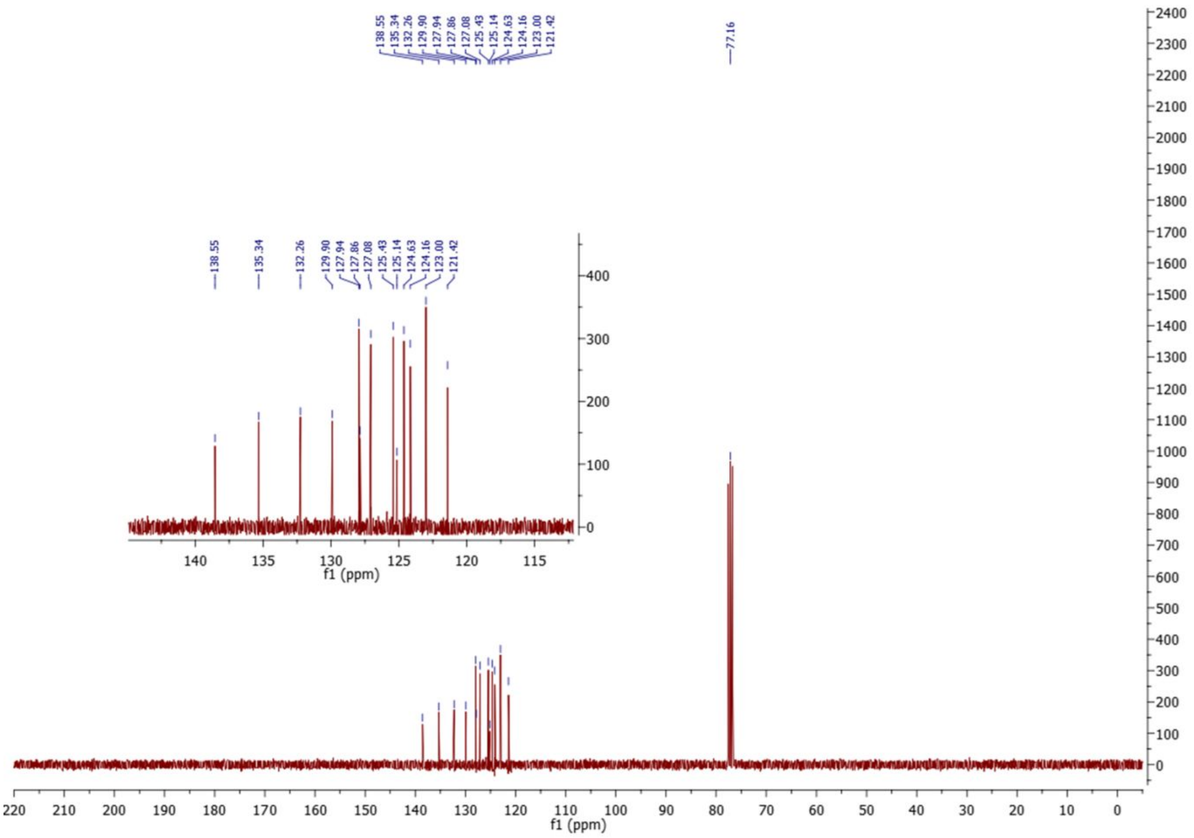

1.8. *Endo*-dithia[7]helicene (**2**) (300MHz, $CDCl_3$)

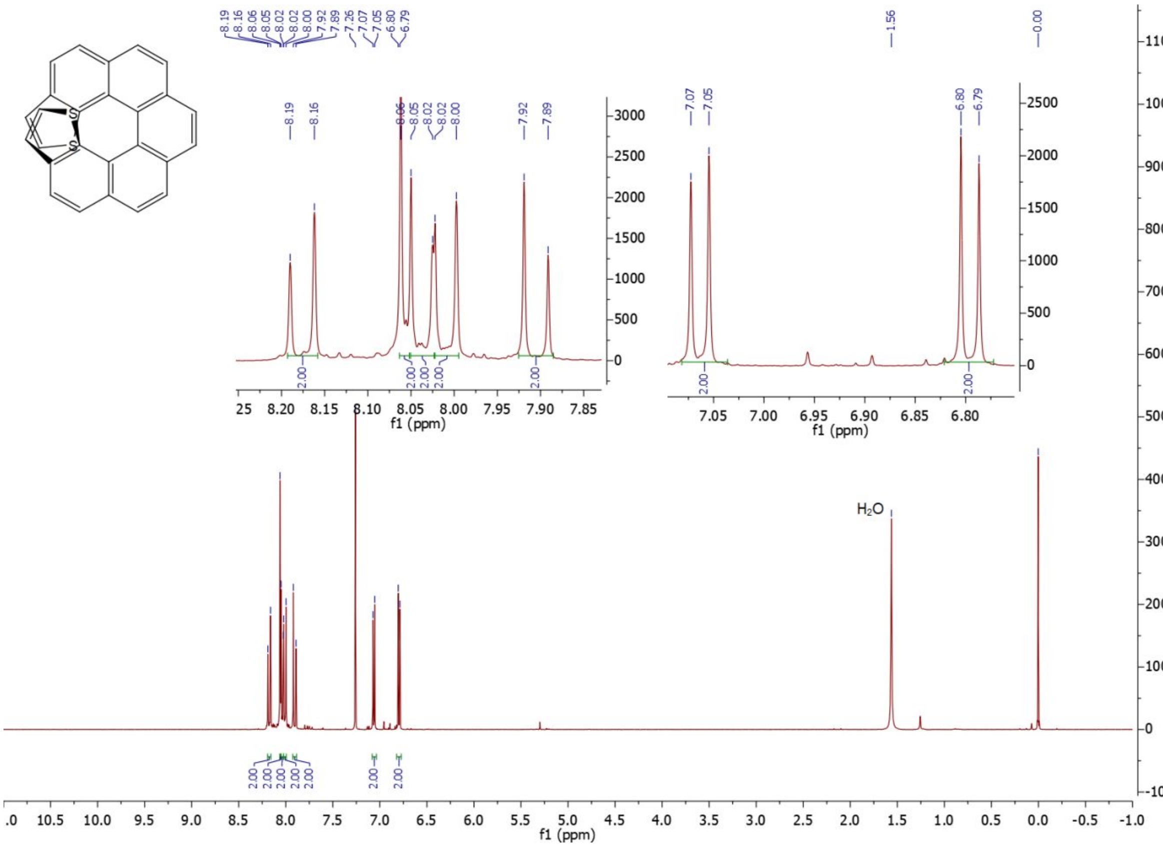

Endo-dithia[7]helicene (**2**) (75MHz, $CDCl_3$)

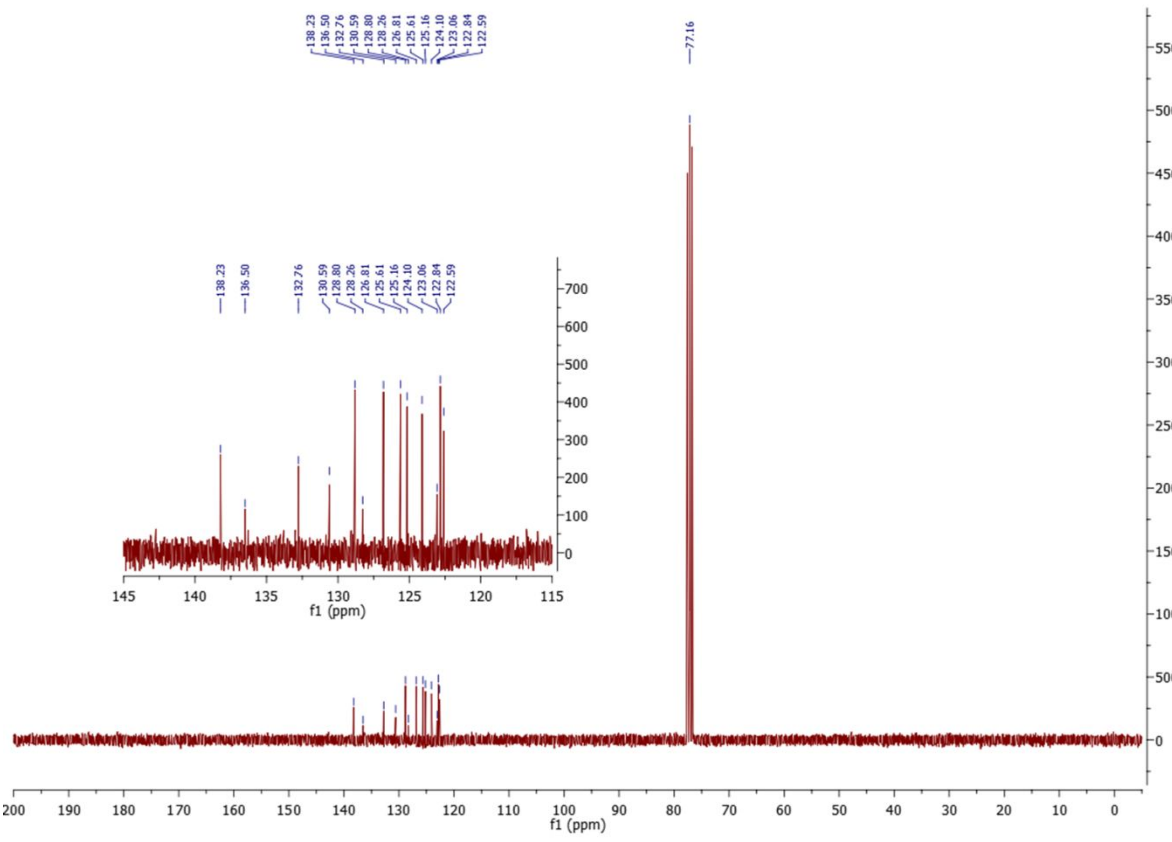

2. Melting point correction

The reported melting points were measured with a melting point apparatus with microscope. To correct the conventional melting point, we used a calibration line made with a set of Melting Points Standards from Merck.

mp standard	mp (lit., ºC)	mp (exp., ºC)
1-Heptadecanocarboxylic acid	69-71	63.3-65.2
Benzoic acid	121-123	116.2-116.9
4-Methoxybenzoic acid	182-184	177.4-179.4
Caffeine	235-237	233-235
Anthraquinone	283-286	280-283

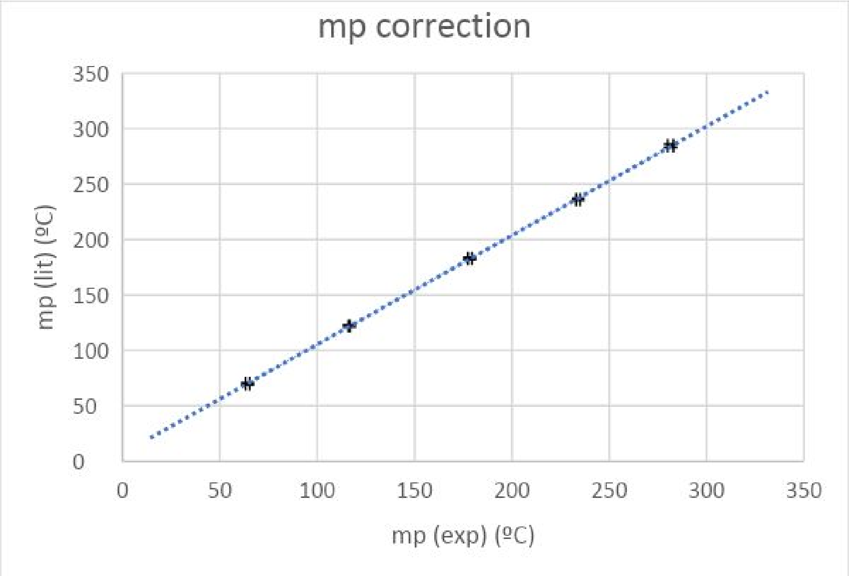

	mp (exp.), °C	mp (corrected), °C
(*E*)-1,2-bis(4-bromophenyl)ethene	191.45	195.34
3,6-dibromophenanthrene	187.65	191.60
3,6-bis((*E*)-2-(thiophen-2-yl)vinyl)phenanthrene	200.05	203.80
3,6-bis((*E*)-2-(thiophen-3-yl)vinyl)phenanthrene	189.50	193.42

3. Experimental UV-vis spectra of 7 and 8

3,6-bis((E)-2-(thiophen-2-yl)vinyl)phenanthrene **(7)**

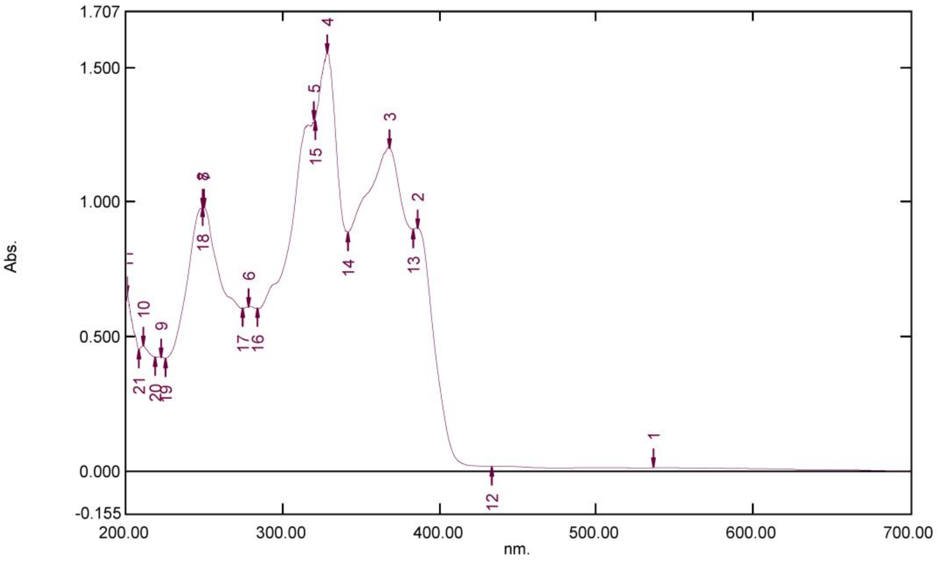

Measurement Properties

Wavelength Range (nm.):	200,00 to 700,00
Scan Speed:	Medium
Sampling Interval:	0,1
Auto Sampling Interval:	Disabled
Scan Mode:	Single

Sample Preparation Properties

Concentration:	5.9·10^{-5} M
Solvent:	n-hexane
Path Length:	10 mm
Additional Information:	25ºC

No.	P/V	Wavelength	Abs.	Description
1	↑	536.50	0.013	
2	↑	385.80	0.904	
3	↑	368.00	1.203	
4	↑	328.10	1.552	
5	↑	320.40	1.302	
6	↑	278.70	0.612	
7	↑	250.10	0.979	
8	↑	248.80	0.978	
9	↑	222.30	0.425	
10	↑	211.20	0.465	
11	↑	201.10	0.655	
12	↓	433.30	0.018	
13	↓	383.40	0.899	
14	↓	341.80	0.887	
15	↓	320.80	1.301	
16	↓	284.50	0.603	
17	↓	274.20	0.604	
18	↓	249.30	0.977	
19	↓	225.20	0.419	
20	↓	218.80	0.423	
21	↓	208.40	0.450	

3,6-bis((E)-2-(thiophen-3-yl)vinyl)phenanthrene **(8)**

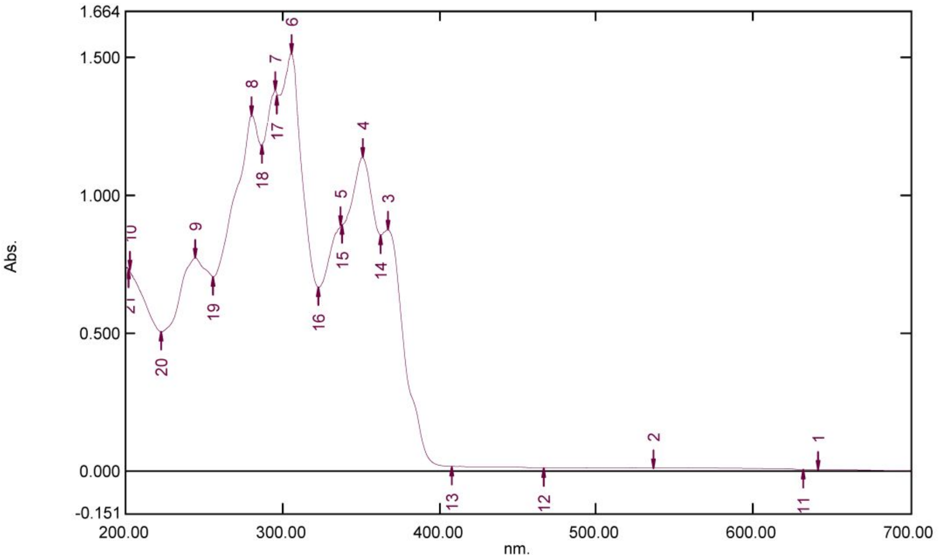

Measurement Properties

Wavelength Range (nm.):	200,00 to 700,00
Scan Speed:	Medium
Sampling Interval:	0,1
Auto Sampling Interval:	Disabled
Scan Mode:	Single

Sample Preparation Properties

Concentration:	$6.6 \cdot 10^{-5}$ M
Solvent:	n-hexane
Path Length:	10 mm
Additional Information:	25ºC

No.	P/V	Wavelength	Abs.	Description
1	↑	641.50	0.005	
2	↑	536.50	0.012	
3	↑	366.90	0.874	
4	↑	350.90	1.136	
5	↑	337.50	0.892	
6	↑	306.00	1.513	
7	↑	295.50	1.377	
8	↑	280.70	1.288	
9	↑	244.40	0.773	
10	↑	202.50	0.732	
11	↓	631.50	0.005	
12	↓	466.60	0.011	
13	↓	407.50	0.017	
14	↓	362.90	0.855	
15	↓	338.10	0.889	
16	↓	322.80	0.664	
17	↓	296.80	1.360	
18	↓	287.00	1.180	
19	↓	256.10	0.703	
20	↓	222.90	0.503	
21	↓	202.10	0.729	

4. DFT and TDDFT Calculations

4.1. Conformational analysis of **7** and **8**

Table S1. Conformational analysis of **7** at the WB97XD/6-311++G(d,p) level of theory in n-hexane as PCM.[a] Structure, energy (*E*) in Ha, relative energy (Δ*E*) in kcal/mol, degeneracy in the equilibrium (*g*) and population in %.[b] Major conformations in entry **3** and **7** are printed in blue and are referred as **7** (C_{2v}).and **7** (C_v) throughout the text.

	**Structure**	***E* (Ha)**	**Δ*E* (kcal/mol)**	*g*	**%**
**1**	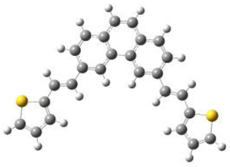	-1797.865627	1.89	1	1.24
**2**	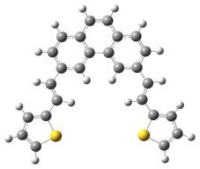	-1797.864123	2.83	1	0.25
**3**	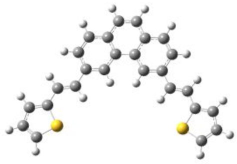	-1797.86864	0	1	30.24
**4**	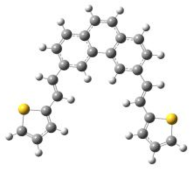	-1797.867131	0.95	1	6.11
**5**	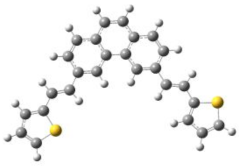	-1797.867256	0.87	2	13.97
**6**	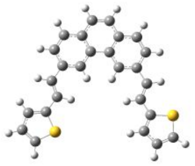	-1797.865788	1.79	2	2.95
**7**	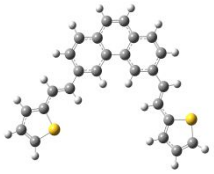	-1797.868066	0.36	2	32.93

**8**	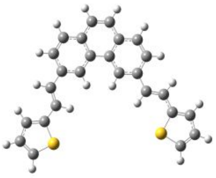	-1797.86638	1.42	2	5.52
**9**	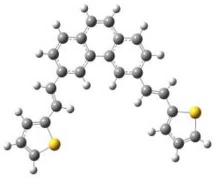	-1797.864945	2.31	2	1.21
**10**	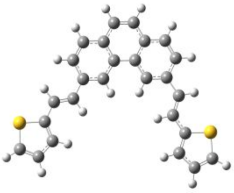	-1797.866387	1.41	2	5.56

Table S2. Conformational analysis of **8** at the WB97XD/6-311++G(d,p) level of theory in n-hexane as PCM.[a] Structure, energy (*E*) in Ha, relative energy (ΔE) in kcal/mol, degeneracy in the equilibrium (*g*) and population in %.[b] Major conformations in entry **1** and **5** are printed in blue and are referred as **8**(C_{2v}).and **8**(C_v) throughout the text.

	**Structure**	***E* (Ha)**	**ΔE (kcal/mol)**	*g*	**%**
1	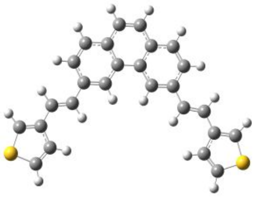	-1797.867455	0	1	31.66
**2**	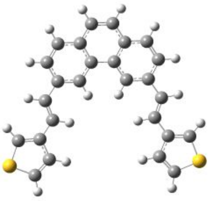	-1797.862751	2.95	1	0.21
**3**	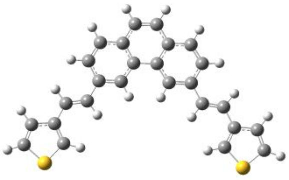	-1797.864236	2.02	1	1.05
**4**	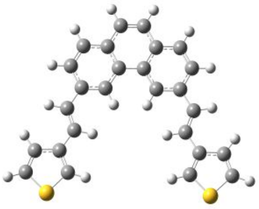	-1797.865915	0.97	1	6.19

5	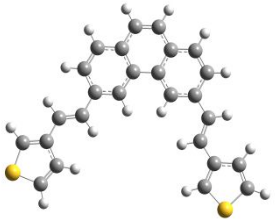	-1797.866825	0.39	2	32.50
6	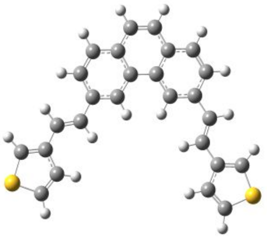	-1797.865196	1.42	2	5.79
7	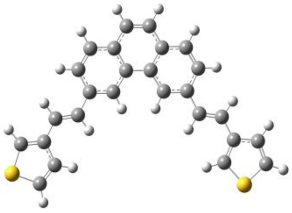	-1797.865984	0.92	2	13.34
8	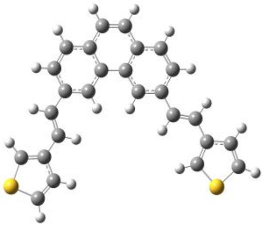	-1797.86353	2.46	2	0.99
9	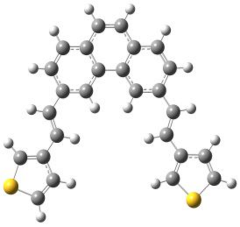	-1797.864485	1.86	2	2.72
10	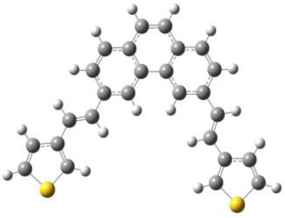	-1797.865154	1.44	2	5.54

[a] All structures were optimized in a planar, fully conjugated conformation, i.e. are either C_{2v} or C_v point group symmetry. It should be noted at this point that, depending on the quantum-chemical method used (functional and base), the located planar stationary state may have either zero or one imaginary frequency. To handle this spurious result, the relevant most important conformations were further optimized to afford true minima with zero imaginary frequencies. These fully optimized geometries are geometrically and energetically essentially indistinguishable from the planar structures, affording nearly indistinguishable spectra. For the sake of simplicity during calculations and confection of Tables

S1-2, we have maintained the original flat structure of the whole set knowing that it would not affect quantitatively the final conclusions.

[b] Populations were calculated based on a Maxwell-Boltzmann distribution of the conformers:

$$N_i/N = g_i e^{-E_i/kT} / \sum_i g_i e^{-E_i/kT}$$

Where 100 N_i/N = %, g_i = is the degeneracy of a given conformation in the overall equilibrium (g_i = 1 for C_{2v} and g_i = 2 for C_v), E_i is the energy of a given conformation, k is the Boltzmann constant and T = 298.15 K.

This conformational outcome is without doubt the combination of many stereoelectronic effects, but can be rationalized as follows. In first place, thiophene seems to prefer its shorter double bond, i.e. C(2)=C(3), eclipsed with the vinyl hydrogen in a s-*trans* type of conformation. This holds both for **7** and **8** in the two preferred conformations (entries in blue). In second place, the vinyl moiety in its attachment to the phenanthrene core slightly prefers a less-congested s-*trans* conformation again, which explains the C_{2v} conformations for **7** and **8**, but only marginally respect to the s-*cis*. It is remarkable that **7**(C_{2v}) and **8**(C_{2v}) (entries 3 in Table S1 and 1 in Table S2) are indeed slightly more stable than the corresponding major conformers **7**(C_s) and **8**(C_s) (entries 7 in Table S1 and 5 in Table S2), although only by the exiguous amount of <0.4 kcal/mol. These last ones are, in turn, the most populated ones since they benefit from the statistical effect of a smaller symmetry number (C_s compared to C_{2v} point group), as explained in the main text.

4.2. Natural Transition Orbitals (NTOs) of the relevant UV-vis bands in **7** and **8**

Natural Transition Orbitals (NTOs) of the relevant bands in **7** and **8**, represented in all cases at the surface isovalue = 0.035. In parenthesis, the major contributing MO to that NTO.

1) NTOs of the vertical transition S_1 of **7** (C_v) (major conformation)

This band at 362.6 nm is described by a major set of NTOs contributing a 78% (NTO-1) and 16% (NTO-2):

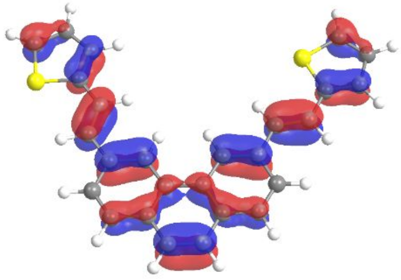

NTO-1 hole (HOMO 78%)

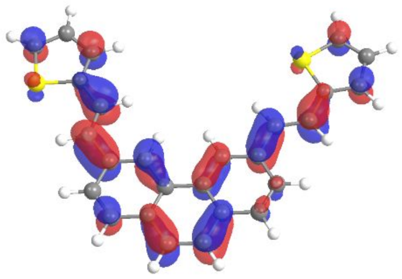

NTO-1 particle (LUMO 78%)

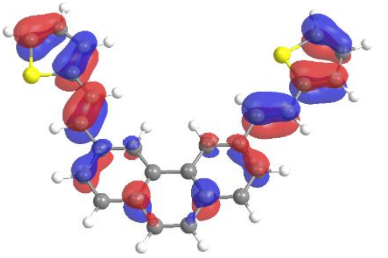

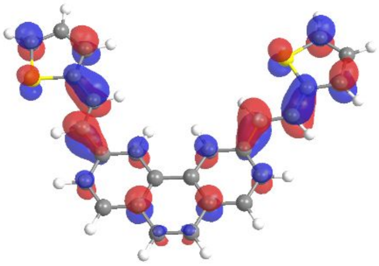

NTO-2 hole (HOMO-1 16%)

NTO-2 particle (LUMO+1 16%)

2) NTOs of the vertical transition S_2 of **7** (C_v) (major conformation)

This band at 321.6 nm is better described by two sets of NTOs contributing a 46% (NTO-1) and another 46% (NTO-2):

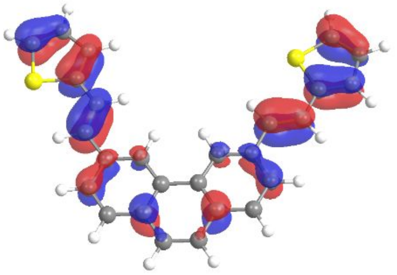

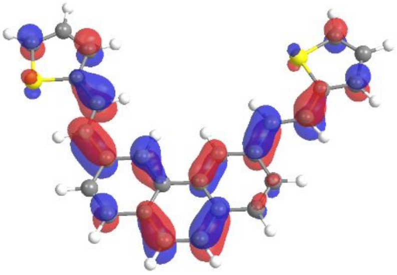

NTO-1 hole (HOMO-1 46%)

NTO-1 particle (LUMO 46%)

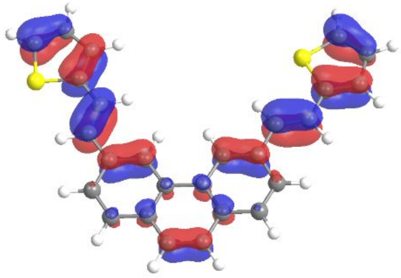

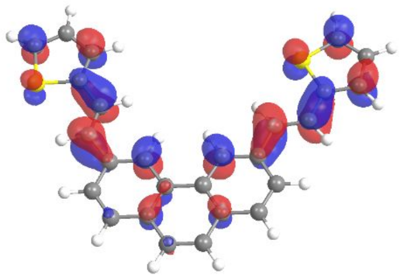

NTO-2 hole (HOMO 40%)

NTO-2 particle (LUMO+1 40%)

3) NTOs of the vertical transition S_1 of **7** (C_{2v}) (minor conformation)

This band at 356.4 nm is described by a major set of NTOs contributing a 77% (NTO-1) and 18% (NTO-2):

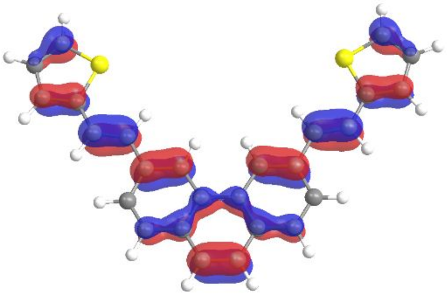

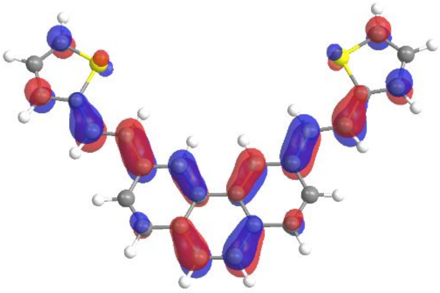

NTO-1 hole (HOMO 77%)

NTO-1 particle (LUMO 77%)

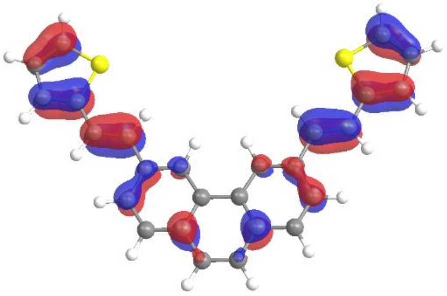

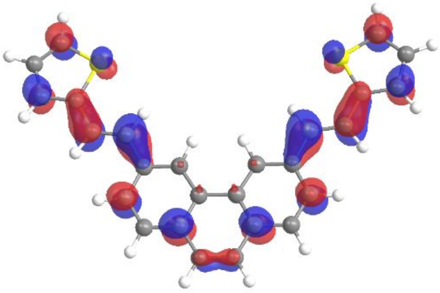

NTO-2 hole (HOMO-1 18%)

NTO-2 particle (LUMO+1 18%)

4) NTOs of the vertical transition S_1 of **8** (C_v)

This band at 344.1 nm is described by a major set of NTOs contributing an 82% (NTO-1) and 10% (NTO-2):

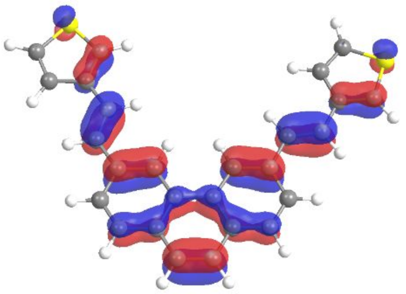

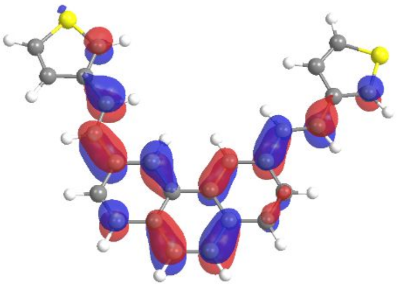

NTO-1 hole (HOMO 82%)

NTO-1 particle (LUMO 82%)

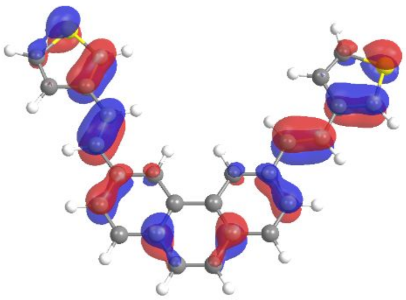

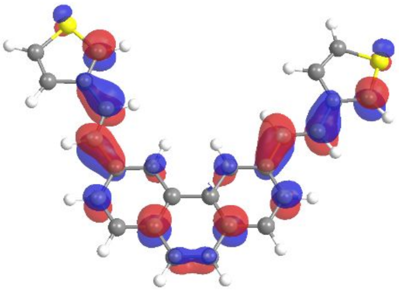

NTO-2 hole (HOMO-1 10%)

NTO-2 particle (LUMO+1 10%)

5) NTOs of the vertical transition S_3 of **8** (C_v)

This band at 295.8 nm is better described by two sets of NTOs contributing a 50% (NTO-1) and another 38% (NTO-2):

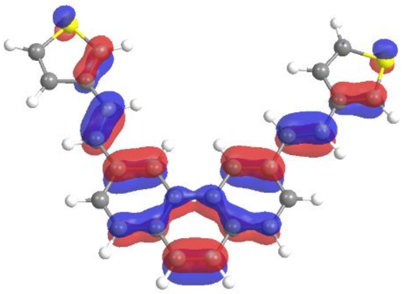

NTO-1 hole (HOMO 50%)

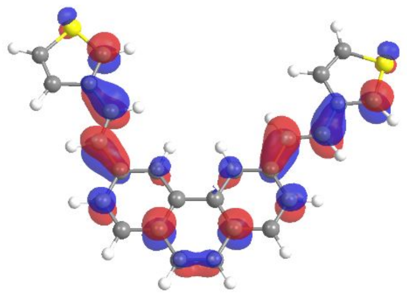

NTO-1 particle (LUMO+1 50%)

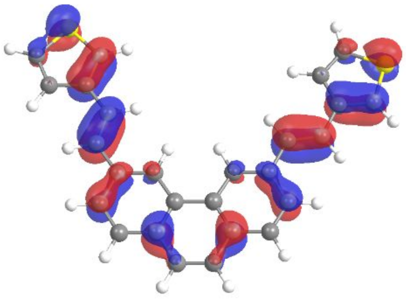

NTO-2 hole (HOMO−1 38%)

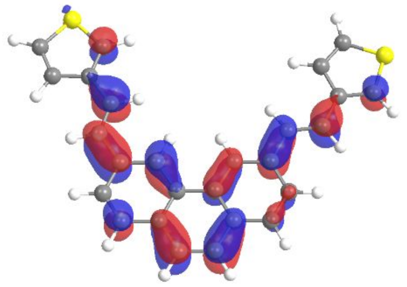

NTO-2 particle (LUMO 38%)

6) NTOs of the vertical transition S_1 of **8** (C_{2v}) (minor conformation)

This band at 336.6 nm is described by a major set of NTOs contributing an 82% (NTO-1) and 11% (NTO-2):

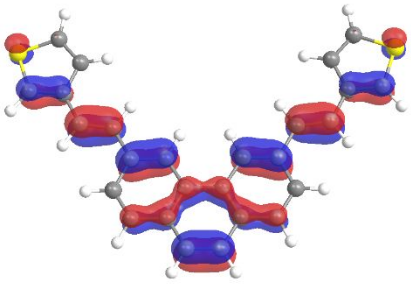

NTO-1 hole (HOMO 82%)

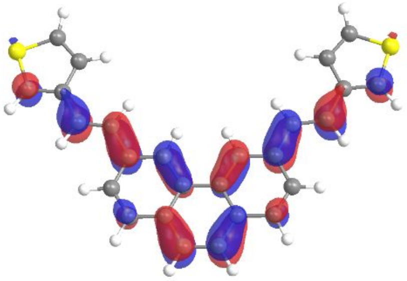

NTO-1 particle (LUMO 82%)

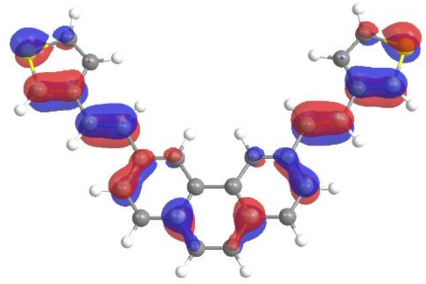

NTO-2 hole (HOMO-1 11%)

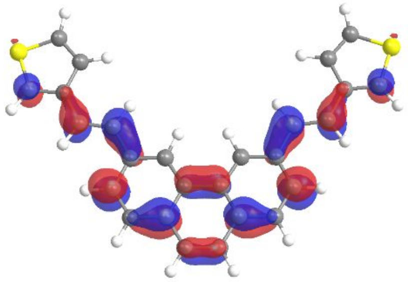

NTO-2 particle (LUMO+1 11%)

4.3. Diagram and Table of energies of the optimized geometries of ground and excited states (*) of **7**, calculated at the WB97XD/6-311++G(2d,2p) level in n-hexane as PCM.

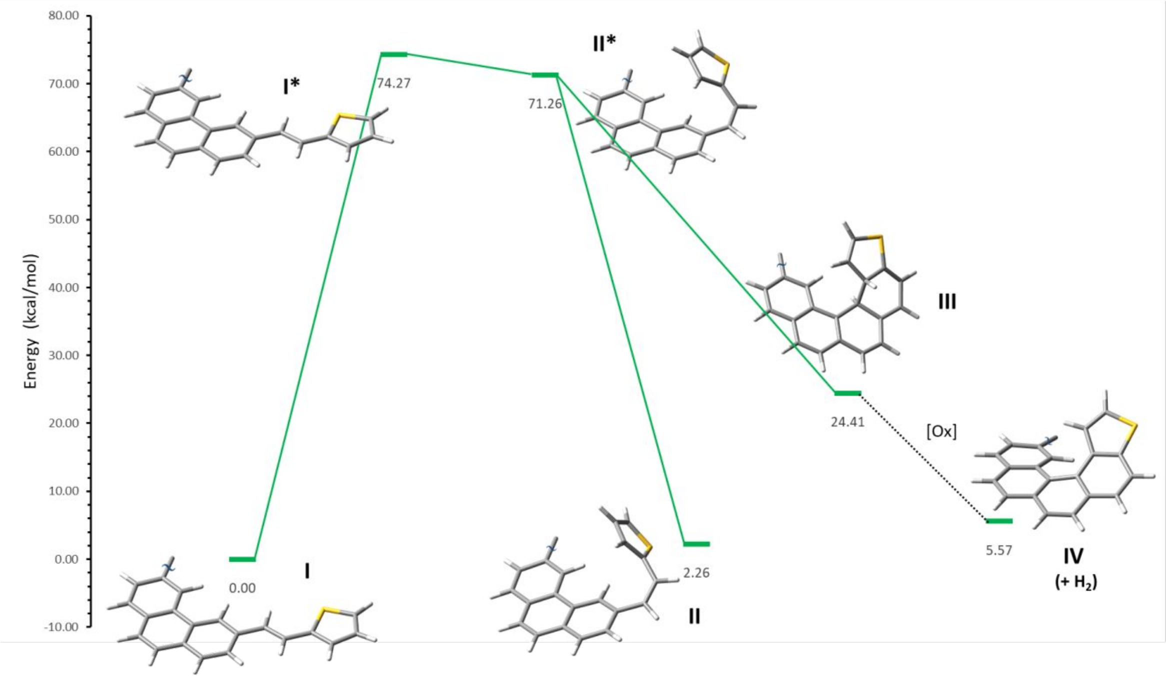

Figure S1. Energy diagram of the photocyclization of **7**, starting with **7** (C2v) (represented in here as **I**) in its route to a thiahelicenes.

Table from Figure S1 above. WB97XD/6-311++G(2d,2p) in n-hexane as PCM

**Molecule**	**Energy (Ha)**	**Relative energy (kcal/mol)**
**I**	-1797.914489	0
**I***	-1797.796128	74.27
**II***	-1797.800936	71.26
**II**	-1797.910885	2.26
**III**	-1797.875586	24.41
**IV**	-1796.728943	5.57 (including H_2)
**H_2**	-1.176668	-

4.4. Optimized geometries of excited states of **7** in S_1**: I***, **II***

Optimized geometries by TDDFT calculations at the WB97XD/6-311++G(2d,2p) level in n-hexane as PCM were followed by analytical vibrational analysis to confirm stationary points (0 imaginary frequencies).

I*

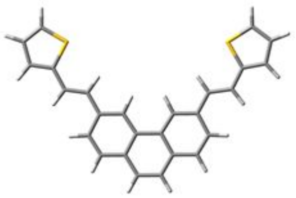

C	3.70788400	-0.85055700	0.00000000
C	3.92985800	-2.25288700	0.00000000
C	2.89673800	-3.14056100	0.00000000
C	1.55544900	-2.68532600	0.00000000
C	1.33496500	-1.30123300	0.00000000
C	2.36509100	-0.37252800	0.00000000
C	-0.69340000	-7.16120800	0.00000000
C	-0.70558400	-5.78495700	0.00000000
C	0.44168800	-4.92209700	0.00000000
C	0.41797800	-3.57048800	0.00000000
C	-2.97525000	-6.80285400	0.00000000
H	4.95131200	-2.61167000	0.00000000
H	3.11148500	-4.19994700	0.00000000
H	1.39050000	-5.44574800	0.00000000
H	-0.54872400	-3.07589100	0.00000000
H	-4.04015800	-6.96239400	0.00000000
C	-0.66617200	3.53270200	0.00000000
C	-1.05733200	4.86309500	0.00000000
C	-2.38835400	5.31220300	0.00000000
C	-2.83989200	6.63409600	0.00000000
C	-4.87284600	5.53073800	0.00000000
H	-0.30654100	5.64367000	0.00000000
H	-5.93299300	5.34100500	0.00000000
C	2.13418300	1.07855100	0.00000000
C	0.87719000	1.64145500	0.00000000
C	0.65497100	3.04381100	0.00000000
C	1.80926300	3.89059900	0.00000000
H	1.69054000	4.96468500	0.00000000
C	3.05619300	3.35941800	0.00000000
H	3.92176900	4.00983600	0.00000000
C	3.27488900	1.94972500	0.00000000
C	4.78248500	0.05479600	0.00000000
C	4.56713900	1.41844600	0.00000000
H	5.41068700	2.09678400	0.00000000
H	5.79276000	-0.33339000	0.00000000
H	-1.44843700	2.78011300	0.00000000
S	-3.76419800	4.22182300	0.00000000
S	-2.34572900	-5.20465500	0.00000000
C	-4.23445200	6.74655400	0.00000000
C	-1.98391700	-7.73735600	0.00000000
H	-2.17228100	-8.80015100	0.00000000
H	-4.76535500	7.68658600	0.00000000
H	-2.16017900	7.47323500	0.00000000
H	0.00000000	1.00925200	0.00000000
H	0.30728000	-0.96485100	0.00000000
H	0.22456400	-7.73062400	0.00000000

II*

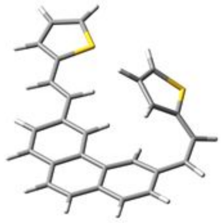

C	3.93912000	-1.67273600	-0.00213900
C	5.16262600	-0.93225800	-0.07016700
C	5.17224200	0.40090900	-0.31177200
C	3.95217700	1.12430100	-0.51001100
C	2.74347200	0.36217200	-0.54780800
C	2.71689000	-0.99661000	-0.23660100
C	1.54296200	2.11749100	1.19423100
C	1.68060100	3.06429600	0.14865200
C	2.81098600	3.34477700	-0.61533000
C	3.95631200	2.51704800	-0.65405500
C	-0.63738400	2.88493300	1.06646600
H	6.09304400	-1.46242100	0.08875300
H	6.10722500	0.94660400	-0.32970300
H	2.83053300	4.26963700	-1.18111800
H	4.91017600	2.99118500	-0.86107500
H	-1.68718000	3.01675400	1.27021700
C	-2.21628300	-1.06925200	-0.17885400
C	-3.45605500	-1.56347800	-0.08418200
C	-4.68274200	-0.79388700	-0.12733300
C	-5.95480900	-1.29286500	-0.04786300
C	-6.44413800	0.95554900	-0.24502700
H	-3.60913200	-2.62975700	0.03746000
H	-6.97253900	1.89072500	-0.31975400
C	1.47556600	-1.74250700	-0.14881800
C	0.22250400	-1.11912100	-0.23547700
C	-0.96439800	-1.81839000	-0.11848200
C	-0.90214300	-3.21815900	0.08079900
H	-1.81143100	-3.79649200	0.16844100
C	0.30340400	-3.84999200	0.18523400
H	0.34083700	-4.91841300	0.35660500
C	1.52091400	-3.13530400	0.09406300
C	3.93699700	-3.06327000	0.24272700
C	2.77336500	-3.77693300	0.27515500
H	2.79191800	-4.84382300	0.45663600
H	4.88433900	-3.56472700	0.39544800
H	-2.09266900	0.00254600	-0.30587100
S	-4.72599700	0.93364300	-0.28711500
S	0.13249800	3.84094600	-0.14516700
C	-6.96057200	-0.29478200	-0.11559200
C	0.23093800	2.03046000	1.68587900
H	-0.06793500	1.36847900	2.48462000
H	-8.01951900	-0.49828900	-0.07032000
H	-6.15666200	-2.34910600	0.05538300
H	0.16747700	-0.04694600	-0.36353000
H	1.86122900	0.83289800	-0.94552400
H	2.39096900	1.62339400	1.63767300

4.5. Diagram and Table of energies of the optimized geometries of ground and excited states (*) of **8**, calculated at the WB97XD/6-311++G(2d,2p) level in n-hexane as PCM.

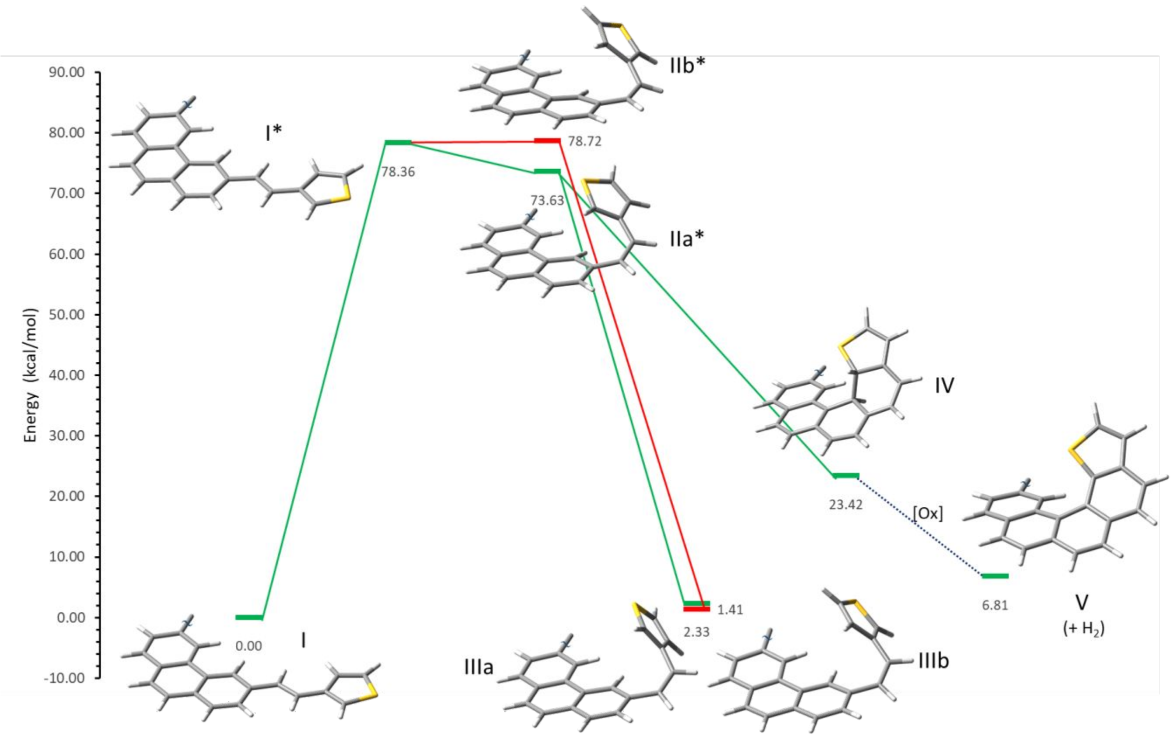

Figure S2 (Figure 4 in main text). Energy diagram of the photocyclization of **8**, starting with **8** (C2v) (represented in here as **I**) in its route to a thiahelicenes

Table from Figure 4 (main text and above). WB97XD/6-311++G(2d,2p) in n-hexane as PCM

**Molecule**	**Energy (Ha)**	**Relative energy (kcal/mol)**
**I**	-1797.913615	0
**I***	-1797.788738	78.36
**IIa***	-1797.796274	73.63
**IIb***	-1797.788164	78.72
**IIIa**	-1797.909906	2.33
**IIIb**	-1797.911373	1.41
**IV**	-1797.876300	23.42
**V**	-1796.726088	6.81 (including H_2)
**H_2**	-1.176668	-

4.6. Optimized geometries of excited states of **8** in S_1**: I***, **IIa***, **IIb***

Optimized geometries by TDDFT calculations at the WB97XD/6-311++G(2d,2p) level in n-hexane as PCM were followed by analytical vibrational analysis to confirm stationary points (0 imaginary frequencies).

I*

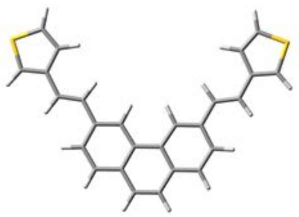

C	0.00000000	1.41043100	3.55472700
C	0.00000000	2.82047400	3.55797100
C	0.00000000	3.53547600	2.39365700
C	0.00000000	2.87245900	1.14735600
C	0.00000000	1.49075400	1.14052700
C	0.00000000	0.72709600	2.32119600
C	0.00000000	4.96676200	-2.89151800
C	0.00000000	5.58998300	-1.60189300
C	0.00000000	4.90787900	-0.31207400
C	0.00000000	3.58398000	-0.13474000
H	0.00000000	3.33736100	4.50928900
H	0.00000000	4.61550900	2.43693200
H	0.00000000	5.56473400	0.55023000
H	0.00000000	2.94079500	-1.00823300
C	0.00000000	-3.58398000	-0.13474000
C	0.00000000	-4.90787900	-0.31207400
C	0.00000000	-5.58998300	-1.60189300
C	0.00000000	-4.96676200	-2.89151800
H	0.00000000	-5.56473400	0.55023000
C	0.00000000	-0.72709600	2.32119600
C	0.00000000	-1.49075400	1.14052700
C	0.00000000	-2.87245900	1.14735600
C	0.00000000	-3.53547600	2.39365700
H	0.00000000	-4.61550900	2.43693200
C	0.00000000	-2.82047400	3.55797100
H	0.00000000	-3.33736100	4.50928900
C	0.00000000	-1.41043100	3.55472700
C	0.00000000	0.67489800	4.78159500
C	0.00000000	-0.67489800	4.78159500
H	0.00000000	-1.22529900	5.71366100
H	0.00000000	1.22529900	5.71366100
H	0.00000000	-2.94079500	-1.00823300
C	0.00000000	-5.85862600	-3.91198000
C	0.00000000	5.85862600	-3.91198000
H	0.00000000	5.65525600	-4.96927800
H	0.00000000	-5.65525600	-4.96927800
H	0.00000000	-3.89924300	-3.05015700
H	0.00000000	-0.99303100	0.18104300
H	0.00000000	0.99303100	0.18104300
H	0.00000000	3.89924300	-3.05015700
C	0.00000000	6.95365400	-1.71122600
C	0.00000000	-6.95365400	-1.71122600
H	0.00000000	7.67119000	-0.90746300
H	0.00000000	-7.67119000	-0.90746300
S	0.00000000	-7.48020400	-3.34110100
S	0.00000000	7.48020400	-3.34110100

IIa*

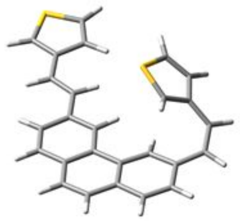

C	3.91722800	-1.69089200	-0.09823800
C	5.17290200	-1.02967600	-0.29903800
C	5.23993500	0.27748200	-0.65009000
C	4.05306500	1.05733400	-0.81097400
C	2.79690100	0.37073100	-0.70957600
C	2.72035700	-0.97386500	-0.32062500
C	0.67521200	3.73764000	-0.05146600
C	1.95652400	3.08368300	-0.00512300
C	3.01383800	3.30988800	-0.89880100
C	4.11350100	2.43915900	-1.03476800
H	6.08123500	-1.60109900	-0.15616800
H	6.20006000	0.76237200	-0.77430400
H	3.00102400	4.23083700	-1.47043200
H	5.05956000	2.85226000	-1.36882800
C	-2.21827100	-0.85521700	-0.25193800
C	-3.47016000	-1.28808400	-0.07339700
C	-4.67133200	-0.47329800	-0.21792600
C	-4.71740000	0.90834500	-0.59404100
H	-3.64811100	-2.32060400	0.20478600
C	1.45338600	-1.65437700	-0.14889900
C	0.22055800	-0.99490600	-0.28774500
C	-0.98910800	-1.63612200	-0.10964500
C	-0.97729600	-3.01249500	0.21959200
H	-1.90677500	-3.54682000	0.35838500
C	0.20382200	-3.67815200	0.37449000
H	0.20252300	-4.72853900	0.63703500
C	1.44641600	-3.02361900	0.20702100
C	3.86506400	-3.05664800	0.26256700
C	2.67490500	-3.70631900	0.40501400
H	2.65221600	-4.75379900	0.67672800
H	4.79558700	-3.58879300	0.41502700
H	-2.06000200	0.18312400	-0.52353900
C	-5.97810700	1.40323200	-0.64495700
C	-0.19896300	3.27937500	0.86773900
H	-1.22393700	3.57706200	1.01307100
H	-6.28779800	2.40225000	-0.90056200
H	-3.84687100	1.50485800	-0.82073500
H	0.20492900	0.06100900	-0.51888500
H	1.92981500	0.85657500	-1.12397700
H	0.42896000	4.49270300	-0.78354400
C	1.98355800	2.12177000	1.03957600
C	-5.92787600	-0.96811700	0.00136900
H	2.86287500	1.67853500	1.47127600
H	-6.19062600	-1.97125700	0.29370900
S	-7.14777800	0.21025200	-0.23927900
S	0.49736300	2.05401200	1.88616800

IIb*

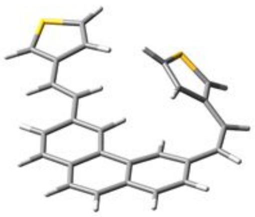

C	4.06571500	-1.70536300	0.04660500
C	5.29671900	-0.96992300	-0.00991500
C	5.31786300	0.36054700	-0.26129200
C	4.10515100	1.09158500	-0.48512300
C	2.89216600	0.33347600	-0.52464100
C	2.84503500	-1.00741200	-0.20149700
C	1.54886000	2.35361700	1.09554900
C	1.78748200	3.19914100	-0.05940300
C	3.01895300	3.34392600	-0.74194200
C	4.12652000	2.47796100	-0.68001400
H	6.22141500	-1.50556100	0.16358500
H	6.25761900	0.89830900	-0.27503900
H	3.13250200	4.24283300	-1.33912300
H	5.09918400	2.91937900	-0.87775500
C	-2.09033100	-1.03643000	-0.14399700
C	-3.34095600	-1.51802400	-0.09906900
C	-4.55048000	-0.71048000	-0.14387700
C	-4.61182200	0.72047900	-0.20904000
H	-3.50304300	-2.58744800	-0.02687800
C	1.58934300	-1.74385700	-0.11106800
C	0.34901300	-1.11052600	-0.18636700
C	-0.85299200	-1.79944600	-0.08652300
C	-0.80333700	-3.20376700	0.09062100
H	-1.71847200	-3.77448100	0.16640300
C	0.39458100	-3.84828100	0.19215800
H	0.42070800	-4.91927500	0.34914000
C	1.62411600	-3.14492600	0.11702100
C	4.04363000	-3.08484600	0.28401800
C	2.86181700	-3.79393700	0.30048800
H	2.87291000	-4.86210700	0.47465300
H	4.98181100	-3.60137000	0.44292000
H	-1.95137300	0.03649900	-0.22975500
C	-5.87997900	1.19651700	-0.23880400
C	0.27128400	2.39705000	1.53756200
H	-0.16069100	1.87899000	2.37671600
H	-6.20106300	2.22311800	-0.28634300
H	-3.74633200	1.36546700	-0.23070400
H	0.30401300	-0.03539300	-0.29709600
H	1.99926900	0.83402400	-0.85863300
H	2.32407900	1.78094000	1.57875100
C	0.61930100	3.88102200	-0.40408800
C	-5.80565100	-1.25854700	-0.12550800
H	0.48733400	4.56388500	-1.22654900
H	-6.05810900	-2.30497500	-0.08024600
S	-7.03936100	-0.07290600	-0.18802800
S	-0.69149800	3.46852000	0.59208100

4.7. Natural Transition Orbitals (NTOs) of the geometrically optimized S_1 excited state of **7**

Natural Transition Orbitals (NTOs) of the absolute minimum **II***, represented at the surface isovalue = 0.035.

II* is described by a single NTO contributing a 95% (NTO-1) of composition: H $\rightarrow$ L (91%); H−1 $\rightarrow$ L (4%). Notice the σ-bonding interaction between C(2) of thiophene and C(4) of phenanthrene.

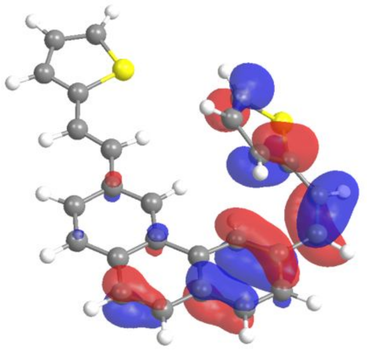

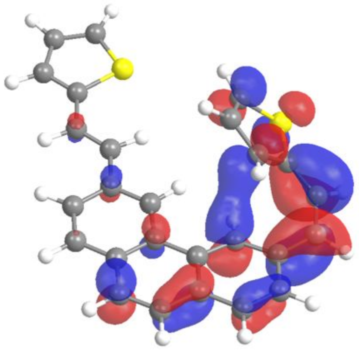

NTO-1 hole NTO-1 particle

4.8. Natural Transition Orbitals (NTOs) of the geometrically optimized S_1 excited state of **8**

Natural Transition Orbitals (NTOs) of the absolute minimum **IIa***, represented at the surface isovalue = 0.035.

IIa* is described by a single NTO contributing a 97% (NTO-1) of composition: H $\rightarrow$ L (90%); H−1 $\rightarrow$ L (4%); H−1 $\rightarrow$ L (3%). Notice the σ-bonding interaction between C(2) of thiophene and C(4) of phenanthrene.

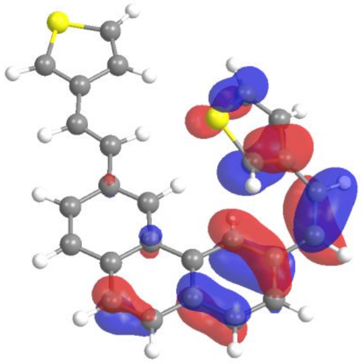

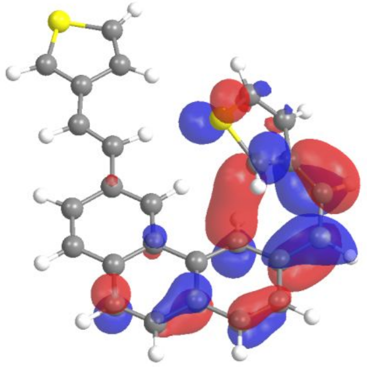

NTO-1 hole NTO-1 particle

Natural Transition Orbitals (NTOs) of the relative minimum **IIb***, represented at the surface isovalue = 0.035.

IIb* is described by a single NTO contributing a 92% (NTO-1) of composition: H $\rightarrow$ L (89%); H−1 $\rightarrow$ L (3%). Notice the lack of σ-bonding interaction between C(2) of thiophene and C(4) of phenanthrene.

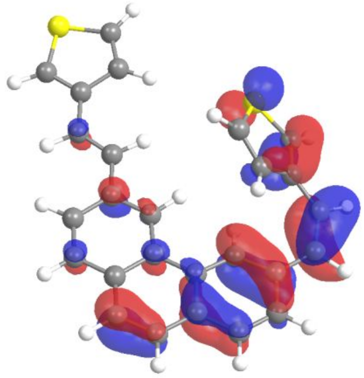

NTO-1 hole

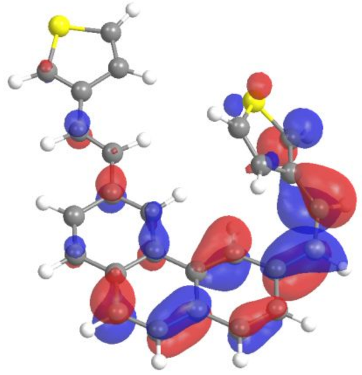

NTO-1 particle
